# Probiogenomics as a Computational Biotechnology Framework: Safety-Gated Genome Analytics for Candidate Probiotic Prioritization and Validation

**DOI:** 10.34133/csbj.0229

**Published:** 2026-09-23

**Authors:** Nattarika Chaichana, Komwit Surachat

**Affiliations:** Department of Biomedical Sciences and Biomedical Engineering, Faculty of Medicine, Prince of Songkla University, Hat Yai, Songkhla 90110, Thailand.

## Abstract

Whole-genome sequencing has transformed probiotic discovery into a strain-resolved computational problem, but genome data alone cannot establish probiotic efficacy, complete safety, or regulatory acceptability. This review frames probiogenomics as a safety-gated decision-support framework for candidate probiotic prioritization and validation. The framework integrates strain provenance, genome quality, strain authentication, taxonomic confidence, antimicrobial resistance screening, virulence and toxin assessment, plasmid, prophage and mobile-element analysis, undesirable metabolite screening, functional prediction, comparative genomics, intended-use context, and validation planning. We emphasize that genome-based safety screening should be interpreted as early-stage risk triage under specified tools, databases, thresholds, and genome quality. Candidates with acceptable safety evidence can then be prioritized through pathway-level functional trait mining, comparative and evolutionary interpretation, systems biology, multi-omics, artificial intelligence or machine learning-assisted prioritization, and targeted phenotypic validation. However, predicted genes and pathways should be treated as hypotheses until expression, biological activity, product accumulation, or matched phenotypes are demonstrated under relevant host, product-matrix, dose, exposure-route, and application conditions. The framework is modular rather than one-size-fits-all: Human probiotics, animal feed probiotics, aquaculture probiotics, plant-associated beneficial microbes, starter cultures, dietary supplements, postbiotic source strains, and live biotherapeutic products require different safety questions, validation endpoints, environmental-release considerations, manufacturing controls, and regulatory pathways. We conclude that probiogenomics is most useful when it preserves uncertainty, reports negative and ambiguous findings, uses versioned and reproducible workflows, and links genome-derived predictions to auditable decision rules, an application-dependent evidence continuum, and context-specific validation.

## Highlights

• Defines probiogenomics as a safety-gated and reproducible computational biotechnology framework for candidate probiotic prioritization.
• Proposes a decision-oriented genome analytics model that separates strain authentication, risk screening, functional prediction, and validation.
• Maps core bioinformatics tools and database-dependent decisions to auditable workflow outputs and reporting requirements.
• Emphasizes cautious claim language: genome-predicted traits are hypotheses until molecular, phenotypic, or application-relevant validation is provided.

## Introduction

In this review, probiogenomics is treated as a genome-resolved, systems-oriented, and decision-oriented framework rather than a new discipline or a substitute for classical probiotic testing. The framework is primarily developed for bacterial candidate probiotic strains and related bacterial microbial products because several core modules, including antimicrobial resistance (AMR) screening, plasmid and prophage analysis, virulence assessment, and mobile genetic element (MGE) interpretation, are bacterial-centric. Although some principles may extend to yeasts and other microbial candidates, these applications require organism-specific safety markers, genome features, functional assays, and regulatory interpretation. A genome is used to make auditable decisions about identity, safety, functional plausibility, comparative context, intended-use suitability, regulatory relevance, and validation priority. This framing is necessary because strains specifically being evaluated for probiotic application, but supported only by genome-based screening or preliminary phenotypic evidence, should be referred to as candidate probiotic strains, whereas the term probiotic should be reserved for strains with demonstrated benefit in the relevant host, dose, and intended-use context [1–4]. The framework is built on 3 interpretive principles. First, a newly isolated or sequenced microorganism should be described as a candidate probiotic strain only when it is specifically being evaluated for probiotic application; otherwise, intended-use-specific terms such as starter-culture candidate, plant-associated beneficial microbe, aquaculture candidate, postbiotic source strain, or live biotherapeutic candidate should be used. Second, genome-predicted traits should be treated as hypotheses until expression, biological activity, product accumulation, or matched phenotypes are confirmed. Third, genome-based safety screening identifies known risk signals under specified tools, databases, thresholds, and genome quality, but does not prove complete safety, probiotic efficacy, GRAS (Generally Recognized as Safe) or QPS (Qualified Presumption of Safety) suitability, clinical acceptability, or regulatory approval. These principles are introduced here to reduce repetition in later sections and to keep subsequent discussions focused on specific decision points. Consensus and guideline documents define probiotics by demonstrated health benefit, whereas many genomic studies operate earlier in the discovery pipeline, where evidence is limited to strain identity, predicted traits, and selected *in vitro* phenotypes [1,2]. Regulatory and safety interpretation should therefore be separated from genome-based screening because evidence requirements differ across food, feed, dietary supplement, live biotherapeutic, starter-culture, animal, aquaculture, plant-associated, and postbiotic applications. These intended-use and regulatory contexts are summarized in Table 4. QPS should be understood as a European Food Safety Authority (EFSA) taxonomic-unit pre-assessment framework with relevant qualifications, not as strain-level market authorization or universal probiotic safety. GRAS should be interpreted as a use-specific safety conclusion under defined food-use, exposure, identity, manufacturing, and specification conditions, not as generic approval. Live biotherapeutic products require defined strain identity, manufacturing and quality control, stability, dose, target-population safety, clinical evidence, and regulatory review [5–9].

Strain authentication is the first computational checkpoint because downstream safety and function interpretation depend on the correct biological frame. Here, strain authentication means genome-resolved confirmation that the candidate matches the declared species and strain and is not mislabeled, contaminated, mixed, or taxonomically ambiguous [3,4,10]. Average nucleotide identity (ANI), standardized genome taxonomy, and phylogenomics provide higher resolution than 16S ribosomal RNA (rRNA) or species labels alone, especially in complex or recently revised taxa [4,10–12]. In addition, safety screening should follow authentication and evaluate acquired AMR genes, virulence-associated loci, toxin genes, plasmids, prophages, insertion sequences, and other MGEs within the intended host, exposure route, product matrix, dose, manufacturing process, and jurisdiction [13–21]. Therefore, authentication and safety screening determine whether a candidate is suitable for functional prioritization or should pause, be excluded, or be restricted to a specific intended use.

Functional mining should convert genome content into testable hypotheses and pathway-level interpretations rather than be interpreted as direct evidence of probiotic function. Annotation, orthology inference, bacteriocin and biosynthetic gene cluster (BGC) prediction, carbohydrate-active enzyme (CAZyme) analysis, pathway reconstruction, and comparative genomics can nominate traits related to gastrointestinal survival, antimicrobial activity, exopolysaccharide (EPS), γ-aminobutyric acid (GABA) or vitamin production, and carbohydrate utilization [11,22–27]. However, modern probiogenomics should move beyond isolated gene presence by considering pathway completeness, regulatory context, metabolic network structure, strain persistence, microbial interactions, and condition-dependent expression. Published applications show that probiogenomics is already being used across diverse candidate probiotic taxa, including *Lactococcus*, *Lactobacillus*-related lineages, *Bifidobacterium*, *Enterococcus*, *Pediococcus*, *Weissella*, and *Bacillus* [28–44]. These studies collectively show that useful technological or probiotic-associated traits can coexist with genus-, lineage-, or strain-specific safety concerns, reinforcing the need for taxon-aware and intended-use-aware interpretation. Comparative analyses add evolutionary and systems-level context by distinguishing conserved traits from accessory, lineage-associated, mobile, or strain-specific features [45–48]. For candidate probiotics, this distinction is practical: A functional locus shared across a clade, a bacteriocin cluster confined to one strain, and an AMR gene linked to a plasmid-like contig imply different levels of priority, novelty, stability, and risk. Comparative probiogenomics can also reveal adaptive evolution, gene gain and loss, horizontal gene transfer, niche adaptation, host-associated evolution, and fermentation-associated domestication. Thus, comparative genomics should be used to prioritize validation and risk assessment, not to convert gene presence into unsupported probiotic claims.

The contribution of this review is to organize established practices into an integrated safety-gated probiogenomic framework with explicit decision points. Previous reviews and whole-genome sequencing (WGS)-based workflows have addressed genome quality, taxonomy, safety screening, functional annotation, comparative genomics, and candidate prioritization, but these components may be presented as separate analytical outputs rather than as an evidence-linked decision architecture [49–51]. The novelty of the proposed framework is therefore architectural rather than tool-based: Strain authentication and genome quality precede safety assessment, safety evidence is evaluated before functional promotion, and unresolved safety concerns are not offset by attractive predicted functions. The framework further distinguishes genomic detection, biological plausibility, potential mobility, demonstrated mobility, phenotypic activity, and demonstrated benefit while incorporating comparative context, intended use, and regulatory requirements into candidate decisions. These distinctions are summarized in Table S1. Thus, rather than introducing new computational tools, the framework provides an auditable structure for integrating established evidence, preserving uncertainty, and supporting 4 practical outcomes, including advance, pause, exclude, or restrict intended use, while matching claims to the level of evidence actually available.

## Conceptual Evolution of Probiogenomics

**Fig. 1.**
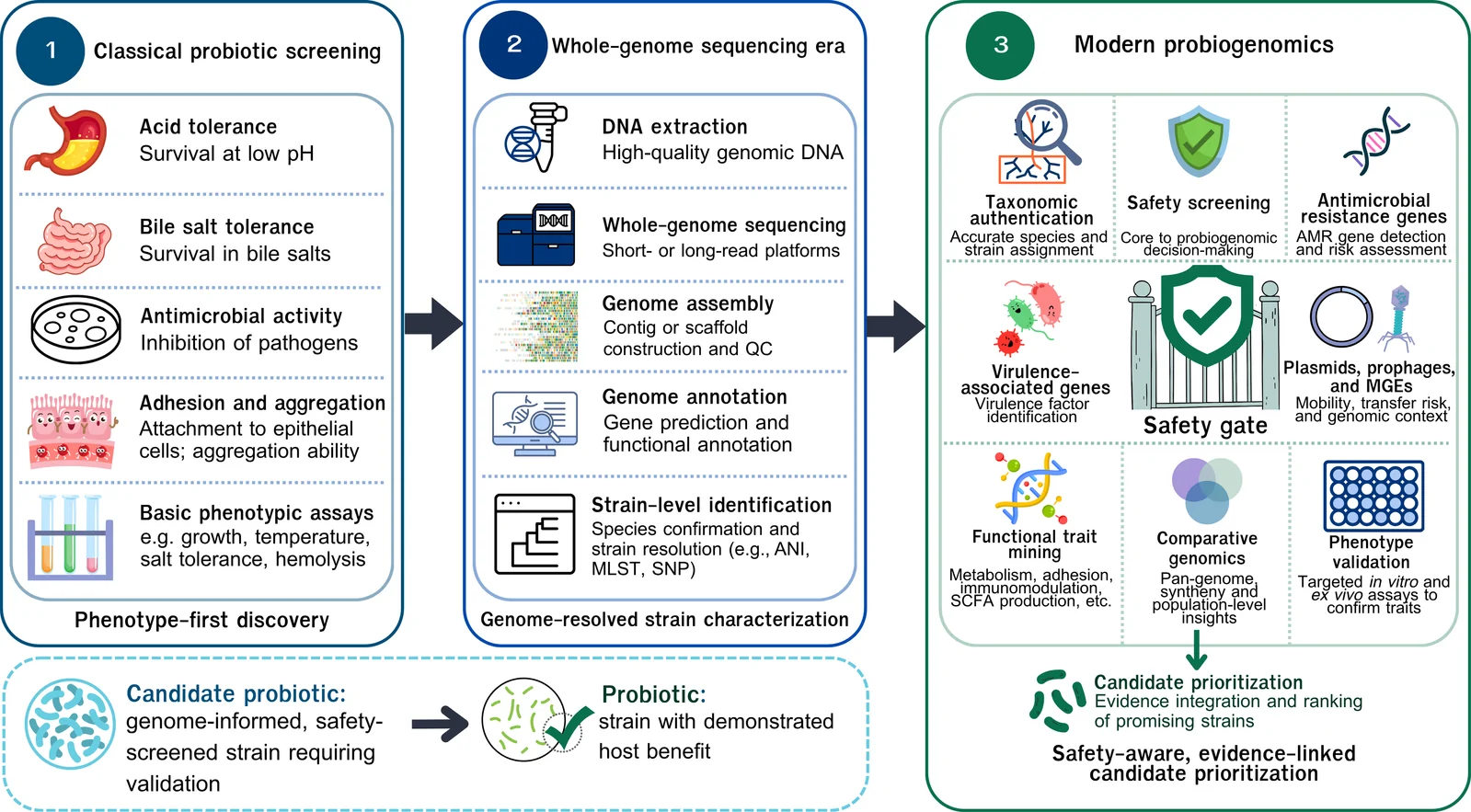
From classical probiotic screening to probiogenomics. Classical probiotic discovery relied mainly on phenotype-first assays, whereas modern probiogenomics integrates genome-based strain authentication, safety screening, functional trait prediction, comparative context, and targeted validation. The figure emphasizes the distinction between a candidate probiotic and a probiotic with demonstrated host benefit.

Probiogenomics reflects a shift from phenotype-first screening to evidence-linked, genome-informed strain prioritization. Classical assays remain essential, but WGS adds identity confirmation, pathway and accessory-gene context, safety screening, and comparison with relevant relatives [49–51] (Fig. 1). In this review, probiogenomics connects strain authentication, genome quality, safety screening, functional hypothesis generation, comparative context, and matched validation through explicit decision points.

This structure prevents probiogenomic studies from becoming simple catalogs of genes with presumed beneficial meaning. Instead, predicted traits should be interpreted through pathway context, expression potential, comparative distribution, intended use, and validation evidence [28,29]. Incomplete pathways, fragmented loci, and unresolved mobile context should remain visible because they define the uncertainty surrounding each candidate [22,23]. Thus, the value of the safety-gated probiogenomic framework is to separate predicted genetic potential, measured phenotype, and demonstrated probiotic benefit, supported by appropriate phenotype-based and host-context validation studies [33,52]. The key concepts used in this review are summarized in Table 1.

**Table 1.** Key concepts and definitions in probiogenomics

Term	Working definition in this review	Practical implication for study design	Supporting sources
Probiotic	Live microorganisms that, when administered in adequate amounts, confer a health benefit on the host.	The term should be reserved for strains with demonstrated benefit in the relevant host, dose, and use context; most newly sequenced strains should be described as candidate probiotics.	[1,2]
Candidate probiotic strain	A strain specifically being evaluated for probiotic application but lacking sufficient evidence of host-relevant health benefit to justify the term probiotic.	This term should not be applied to all newly isolated or sequenced microorganisms. Strains evaluated for other purposes should be described according to their intended use, such as starter-culture candidates, plant-associated beneficial microbes, aquaculture candidates, postbiotic source strains, or live biotherapeutic candidates.	[1,5]
Probiogenomics	A genome-resolved, decision-oriented framework for evaluating candidate probiotics through strain authentication, genome quality assessment, safety screening, functional prediction, comparative context, systems-level interpretation, and validation planning.	Shifts probiotic research from descriptive strain cataloguing toward evidence-linked prioritization and responsible claim development.	[28,29]
Safety-gated probiogenomic framework	A structured framework in which identity, genome quality, and safety-relevant evidence are evaluated before functional traits are promoted.	Prevents attractive predicted functions from overriding unresolved AMR, virulence, toxin, mobile-element, or intended-use concerns.	[6,13]
Genome-predicted trait	A putative trait inferred from genes, pathways, loci, or genome-derived annotations.	Must be reported cautiously; gene presence does not prove expression, pathway activity, product accumulation, biological activity, or host benefit.	[24,25]
Strain authentication	Genome-resolved confirmation that the sequenced culture matches the declared strain and is not mislabeled, mixed, contaminated, or taxonomically ambiguous; distinct from species identification.	Requires species-level confirmation plus strain-level traceability, using ANI, GTDB-Tk, phylogenomics, stable strain ID, accession/provenance records, contamination checks, and SNP or cgMLST analysis where needed.	[3,4,10]
Safety gate	A decision point that classifies candidates as advance, pause, exclude, or restrict intended use based on safety evidence, uncertainty, and intended-use context.	Supports early risk triage and avoids costly validation of strains with unresolved high-confidence safety concerns.	[5,6]
Functional trait mining	Identification and interpretation of genome-predicted traits related to survival, adhesion, antimicrobial activity, EPS, metabolite production, carbohydrate utilization, or host interaction.	Should move beyond single-gene presence toward pathway completeness, regulatory context, genomic neighborhood, comparative distribution, and matched validation.	[22–27,33]
Systems-level interpretation	Interpretation of candidate functionality through pathways, metabolic networks, regulatory interactions, microbial interactions, and host–microbe context rather than isolated genes alone.	Encourages use of pathway reconstruction, network analysis, genome-scale metabolic modeling, and condition-aware validation.	[117,119,120,124,142,143]
Comparative probiogenomics	Use of pangenomes, ANI, Mash, and phylogenomics to interpret whether predicted traits are conserved, accessory, lineage-associated, or strain-specific.	Helps avoid overgeneralizing from a single strain and supports evolutionary interpretation of trait stability, novelty, mobility, and risk.	[45–48]
Multi-omics integration	Integration of genomics with transcriptomics, proteomics, metabolomics, metagenomics, pangenomics, or single-cell omics to test whether predicted traits are active and biologically relevant.	Links genome-derived hypotheses to expression, protein production, metabolite accumulation, strain persistence, community effects, and observed phenotypes.	[117,119,120,124]
AI or ML-guided prioritization	Use of supervised learning, unsupervised learning, genome embeddings, protein language models, graph-based models, or explainable AI to support candidate ranking and functional prediction.	Can assist large-scale prioritization but requires reliable labels, external validation, interpretability, and safety-aware decision rules.	[121–123,136]
Application-dependent evidence continuum	A flexible evidence framework in which genomic prediction may be supported by relevant molecular, phenotypic, host-, application-, product-, clinical-, or regulatory-level evidence according to the intended use and claim.	Presents validation as a continuum rather than a single experimental step and helps match claims to evidence level.	[1,2,33,127]
Regulatory evidence layer	Intended-use-specific interpretation of safety, quality, efficacy, manufacturing, and claim requirements under frameworks such as QPS, GRAS, food or feed use, dietary supplements, and live biotherapeutic products.	Keeps regulatory discussion centralized and prevents genome-based screening from being misinterpreted as regulatory approval.	[5–8,127]
Evidence-linked claim	A statement written at the level directly supported by the available data.	Encourages language such as “genome-predicted”, “putative”, and “candidate probiotic” until stronger evidence is available.	[1,2]

## Recent Advances in Probiogenomic Workflows

**Fig. 2.**
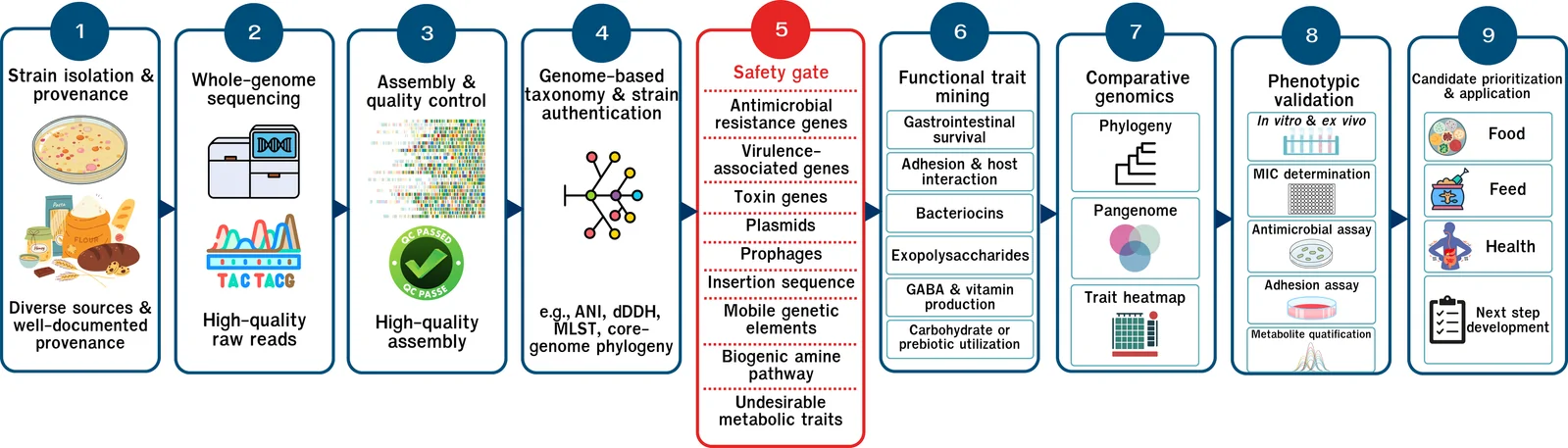
Simplified probiogenomic workflow for candidate strain prioritization. Candidate strains progress from provenance documentation and whole-genome sequencing to assembly quality control, strain authentication, and safety-gated screening. Strains with acceptable safety evidence can then proceed to functional trait mining, comparative genomic analysis, matched phenotypic validation, and application-oriented prioritization.

Recent probiogenomic studies show a clear shift from descriptive genome reports toward integrated decision workflows. The common analytical modules now include strain recovery, sequencing, assembly, annotation, genome-quality assessment, taxonomy, safety screening, functional mining, comparative genomics, and targeted validation. The quality of a study depends less on whether these modules are named and more on whether each module supports a clearly bounded conclusion. The first update is methodological standardization. Tools such as Prokka [26], Bakta [11], eggNOG-mapper [27], CheckM-like quality assessment [53], GTDB-Tk [4], FastANI [3], antiSMASH [24,54], BAGEL4 [25], CAZyme resources [22,55], AMR databases [13,16,56], virulence databases [18], plasmid tools [57,58], prophage tools [21,59], and mobile-element detectors [15] have improved the reproducibility of probiogenomic workflows. However, these tools also make reporting obligations stronger: Software versions, databases, thresholds, assembly quality, and manual curation steps must be visible because they shape the result. A second advance is safety-gated interpretation. Candidate strains are increasingly screened for acquired AMR, virulence-associated genes, toxins, plasmids, prophages, insertion sequences, and other MGEs before functional traits are promoted [13–15]. A third advance is more cautious functional mining: Predicted GABA pathways, bacteriocin clusters, carbohydrate-utilization modules, or adhesion genes should lead to targeted metabolite, inhibition, substrate-use, or host-interface assays rather than broad claims [24,25,34]. A fourth advance is comparative interpretation using pangenomes, phylogenomics, ANI, Mash distances, and trait overlays to classify features as conserved, lineage-associated, niche-associated, accessory, or rare [45–47]. Multi-omics, machine learning, and scoring systems can further support ranking when they remain interpretable and subordinate to safety screening and phenotype validation. Figure 2 summarizes the implementable workflow proposed in this review. Placing the safety gate before functional promotion makes the analysis auditable: A candidate must first be identifiable, assembled well enough to interpret, and screened for safety-relevant signals before genome-predicted functions are used to design validation experiments.

## Genome-Based Taxonomy and Strain-Level Authentication

Genome-based taxonomy is the first analytical checkpoint because it defines the biological and regulatory frame for downstream safety and functional interpretation. A candidate cannot be evaluated responsibly if its species identity, strain designation, or relationship to reference genomes is uncertain. Moreover, misidentification can distort safety interpretation, comparator selection, and species-level claims. Therefore, taxonomy should be treated as an evidence-based result rather than inherited from colony morphology, 16S rRNA similarity, or culture-collection records [1,10,60]. Species identification and strain authentication should be reported as related but distinct checkpoints. Species identification asks whether an isolate belongs to the declared species or taxonomic unit, whereas strain authentication asks whether the sequenced culture corresponds to the declared strain and is traceable, unmixed, uncontaminated, and reproducibly identifiable. In this review, strain authentication refers to genome-resolved confirmation that a candidate isolate corresponds to the declared species and strain, and that it is not mislabeled, contaminated, mixed, or taxonomically ambiguous. ANI, phylogenomics, and standardized genome-taxonomy workflows are useful starting points. ANI supports species-level relatedness, whereas Genome Taxonomy Database (GTDB)-based classification provides a genome-resolved framework when nomenclature has changed or closely related species are difficult to separate [3,4,10,12]. However, species-level ANI or GTDB-Tk classification is not equivalent to strain authentication. Two isolates can belong to the same species but represent different strains with different safety signals, mobile elements, functional loci, or validation histories. These methods determine relevant reference genomes, safety expectations, and whether a trait is common, lineage-associated, or unusual. Authentication also supports product quality because mixed cultures, mislabeling, and ambiguous strain names undermine safety assessment and mechanistic interpretation [30,61]. For research or commercial strains, authentication may require stable strain identifiers, provenance records, culture or accession traceability, contamination and mixed-culture assessment, high-resolution single-nucleotide polymorphism (SNP) analysis, cgMLST, core-genome comparison, or direct comparison with a declared reference genome. However, genome-based taxonomy does not eliminate all taxonomic uncertainty. Recently diverged taxa, species complexes, subspecies-level boundaries, inconsistent nomenclature, and uneven reference-genome quality can complicate interpretation, particularly in groups such as *Lactiplantibacillus*, *Lacticaseibacillus*, *Bifidobacterium*, *Bacillus*, *Enterococcus*, and *Weissella*. Borderline ANI values, discordance between GTDB classification and validly published names, or conflicting phylogenomic placement should be reported as uncertainty rather than forced into a single confident label. This is important because safety expectations, intrinsic resistance interpretation, comparator selection, and probiotic claim language may differ among closely related species or subspecies.

A practical reporting standard should include species assignment supported by ANI, genome taxonomy, or phylogenomics; strain traceability supported by a unique code, deposition, and accession; and comparative placement supported by a tree, distance matrix, or pangenomic context. Borderline or unresolved taxonomy should remain visible as an uncertainty flag because it affects downstream safety gating and functional interpretation [2,5].

## Genome Generation, Assembly, Annotation, and Quality Control

Genome quality defines how confidently downstream safety, functional, and systems-level interpretations can be made. A sequence accession alone does not show whether plasmids, prophages, AMR regions, EPS loci, bacteriocin clusters, or carbohydrate operons are resolved. Draft genomes can support broad taxonomic and gene-presence analyses, but fragmented assemblies may split accessory regions, obscure neighborhoods, and generate false-positive or false-negative interpretations. Contamination, mixed cultures, and misassemblies can also distort ANI, phylogeny, gene calls, and mobile-context inference; therefore, genome quality should be reported before biological claims are made [62].

Assembly strategy should match the intended claim. Short-read assemblies may be sufficient for species confirmation and broad annotation, whereas long-read or hybrid assemblies are more appropriate when claims depend on plasmids, prophages, repeats, AMR localization, bacteriocin clusters, EPS loci, or complete pathway architecture. Long-read assemblies may still require polishing to reduce frameshifts or pseudogene artefacts. Studies making claims about mobile elements, transferable AMR, prophage cargo, or complete biosynthetic loci should report sequencing platform, read type, coverage, assembler or version, polishing strategy, circularization status, and plasmid-resolution evidence [63–66]. Coverage should be reported as contextual evidence rather than as a universal quality threshold, because suitable depth depends on genome size, GC content, repeat structure, platform error profile, read length, strain purity, and the biological claim. Thus, a study focused on taxonomic identity may require less assembly resolution than a study claiming plasmid-borne AMR, prophage cargo, or complete EPS or bacteriocin loci. General assembly reporting should include sequencing platform, read type, depth, assembler, polishing method, genome size, GC content, contig number, N50, completeness, contamination, and accession numbers; however, N50 should be interpreted only as a descriptive contiguity metric and not as a stand-alone indicator of assembly correctness, biological completeness, or safety-critical reliability. A high N50 does not necessarily exclude misassembly, contamination, mixed culture, collapsed repeats, incorrect plasmid reconstruction, or incomplete recovery of accessory regions. Claim-specific reporting should also state whether key regions are complete, fragmented, circularized, plasmid-associated, prophage-associated, MGE-adjacent, or unresolved and whether these interpretations are supported by read-depth distribution, read-to-assembly evidence, taxonomic purity, misassembly checks, plasmid or prophage resolution, and structural consistency of claim-critical loci [31,39,67]. This distinction is important because an assembly sufficient for strain authentication may still be insufficient for strong claims about transferability, genome stability, or pathway architecture. Annotation should be matched to the biological question. General annotation tools such as Prokka and Bakta provide coding-sequence prediction and baseline functional annotation, while orthology-based tools such as eggNOG-mapper support broader functional inference. Specialized databases are needed for safety and functional modules, including AMR tools, virulence databases, plasmid and prophage tools, antiSMASH and BAGEL4 for secondary-metabolite and bacteriocin clusters, and CAZy or dbCAN-type resources for CAZymes [11,22–26]. Because each tool differs in database coverage, detection rules, and false-positive profiles, studies should report software versions, database releases or access dates where relevant, thresholds, and manual-curation steps. Gene calls should also be assessed for identity, coverage, completeness, frameshifts, pseudogene status, genomic context, and taxonomic relevance [68].

Quality control should continue after annotation. Safety hits require review of identity, coverage, gene completeness, genomic neighborhood, and mobility context, whereas functional predictions require pathway completeness and biological plausibility. Database hits should therefore be treated as interpretable evidence rather than final biological conclusions. Negative findings should be reported as no high-confidence hit detected under the specified tools, databases, thresholds, and genome quality, rather than as proof of absence. Table 2 summarizes commonly used computational tools for probiogenomic analysis, including their required inputs, expected outputs, major strengths, key limitations, computational demand, scalability, and recommended applications. This table is intended as a practical guide for selecting tools and interpreting outputs within the safety-gated probiogenomic framework.

**Table 2.** Representative tools, databases, strengths, limitations, computational demand, and expected outputs for probiogenomic analysis

Analytical module	Representative tools or databases	Main input	Expected output	Major strengths	Key limitations	Computational demand and scalability	Recommended application and reporting notes
Read quality control	FastQC, MultiQC [144], fastp [145], Trimmomatic [146]	Raw or quality-filtered Illumina short reads	Read-quality report; trimmed or filtered reads	Rapidly identifies low-quality bases, adapter contamination, sequence duplication, and read-quality problems before assembly.	Does not evaluate assembly correctness, contamination in the final genome, or biological validity of downstream calls.	Low to moderate; suitable for standard desktops or workstations and highly scalable across many samples using batch processing or workflow managers.	Use before short-read assembly and report tool version, filtering parameters, read length, total reads, retained reads, and quality-summary metrics.
Long-read quality control	NanoPlot, NanoFilt, Filtlong, pycoQC	Raw or quality-filtered Nanopore or PacBio long reads	Long-read quality report; quality-filtered long reads	Evaluates read-length distribution, yield, read quality, and filtering performance for long-read or hybrid assembly.	Quality summaries do not confirm circularization, plasmid resolution, or base-level consensus accuracy.	Low to moderate; suitable for desktops or workstations, although large Nanopore or PacBio runs may require additional storage and memory for batch summaries.	Use before long-read or hybrid assembly and report platform, basecalling model where relevant, read N50, yield, coverage, filtering parameters, and retained reads.
Short-read assembly	SPAdes [147], SKESA [148], Unicycler short-read mode [65]	Illumina short reads	Draft short-read genome assembly	Provides accurate base-level assemblies suitable for taxonomic identification, gene detection, and broad safety or functional screening.	Fragmentation may split plasmids, prophages, repeats, EPS loci, bacteriocin clusters, AMR regions, and mobile-element contexts.	Moderate; CPU- and memory-dependent. Large genome collections require batch processing, workflow managers, or HPC resources.	Use when gene detection and broad screening are sufficient. Report assembler, version, k-mer settings if modified, coverage, contig number, N50, genome size, and GC content.
Long-read assembly	Flye [149], Canu [150], Raven [151], Miniasm [152]	Nanopore or PacBio long reads	Long-read draft, closed, or near-complete genome assembly	Improves contiguity and helps resolve plasmids, prophages, repeats, AMR regions, bacteriocin clusters, EPS loci, and mobile elements.	Platform-specific errors may affect frameshifts, pseudogenes, AMR calls, and pathway completeness if polishing is inadequate.	Moderate to high; memory and CPU demand increase with read depth, genome size, repeat complexity, and long-read error profile.	Use when genomic context, circularization, or mobile-element localization is important. Report read N50, coverage, assembler, version, polishing, circularization status, and plasmid-resolution evidence.
Hybrid assembly	Unicycler [65], Trycycler [153], Flye plus short-read polishing [149]	Quality-filtered short reads and long reads	Hybrid assembly with improved contiguity and consensus accuracy	Combines long-read structural resolution with short-read accuracy, supporting stronger interpretation of plasmids, prophages, AMR localization, and complete functional loci.	Requires matched read sets, careful polishing, and additional checks to avoid misassembly or incorrect circularization.	High; requires processing of matched short- and long-read datasets and is best suited to workstations or HPC for large sample sets.	Use when safety or functional claims depend on genomic context. Report short- and long-read coverage, assembly strategy, polishing tools, polishing rounds, circularization, and plasmid or chromosome separation.
Assembly polishing and correction	Pilon [154], Polypolish [155], Medaka, Racon [156], NextPolish [157]	Draft assembly plus reads	Improved consensus sequence	Reduces base-level errors that may affect gene prediction, frameshifts, AMR calls, and pathway completeness.	May not correct structural misassemblies, contamination, or incorrect plasmid or chromosome separation.	Moderate; demand depends on genome size, read depth, and number of polishing rounds. Large-scale polishing benefits from HPC or workflow managers.	Use after long-read or hybrid assembly. Report polishing tool, version, number of polishing rounds, read type used, and whether polishing changed key loci.
Genome quality assessment	CheckM [53], BUSCO [158], QUAST [159], assembly metrics	Draft, polished, or complete genome assembly	Completeness, contamination, genome-size, contiguity, read-support, taxonomic-purity, and assembly-quality metrics	Determines whether the assembly is reliable enough for strain authentication and downstream safety or functional interpretation.	Quality metrics do not guarantee correct taxonomy, correct mobile-element localization, or complete recovery of all accessory regions, absence of misassembly, plasmid resolution, or safety-critical completeness. N50 is only a descriptive contiguity statistic and should not be used alone as evidence of assembly correctness.	Low to moderate; scalable for isolate genomes, but large collections benefit from parallel processing.	Use before biological interpretation. Report completeness, contamination, genome size, GC content, contig count, N50, assembly level, and accession number, but interpret N50 together with read-depth distribution, read-to-assembly support, taxonomic purity, misassembly checks, plasmid or prophage resolution where relevant, and structural consistency of claim-critical loci.
Taxonomic authentication	FastANI [3], GTDB-Tk [4], TYGS and GGDC-based dDDH, core-genome phylogeny	Genome assembly; reference genomes	ANI values; genome-based taxonomy; phylogenetic placement	Confirms strain or species identity and prevents incorrect safety or functional interpretation caused by misclassification.	Borderline ANI, incomplete reference databases, species complexes, and nomenclature differences can complicate interpretation.	Low to moderate for ANI-based screening; moderate to high for large reference-genome comparisons or phylogenomic analyses.	Use before safety and functional analysis. Report reference and type strains, database release, ANI values, TYGS and GGDC method and dDDH values where used, phylogenetic method, and species-boundary criteria.
General genome annotation	Prokka [26], Bakta [11], NCBI PGAP [160]	Genome assembly	Predicted CDS, RNA genes, functional annotations	Provides standardized baseline gene prediction and annotation for downstream safety, functional, and comparative analysis.	Annotations may be generic, incomplete, database-dependent, or inconsistent across tools.	Low to moderate per genome; scalable with parallel processing, although standardized PGAP-style workflows may require more time and controlled environments.	Use as the baseline annotation layer. Report tool version, database version, genetic code, annotation source, and whether annotations were manually curated.
Orthology and functional inference	eggNOG-mapper [27], COG [161], KEGG [142], InterProScan [162]	Predicted proteins or genome annotation	Ortholog groups; functional categories; pathway annotations	Supports interpretation of metabolism, stress response, transporters, carbohydrate use, and putative probiotic-associated functions.	Functional assignments may not confirm gene expression, pathway completeness, protein activity, or phenotype.	Moderate; demand increases with protein set size, database size, and number of genomes. Large-scale analyses benefit from HPC.	Use for pathway-level hypothesis generation. Report database version, annotation thresholds, e-value or bit-score criteria where relevant, and whether pathway completeness was manually reviewed.
AMR screening	AMRFinderPlus [13], CARD and RGI [16], ResFinder [56]	Genome assembly or predicted proteins	AMR genes, resistance-associated mutations, confidence scores	Detects acquired AMR determinants and resistance-associated mutations relevant to safety-gated prioritization.	Partial genes, intrinsic genes, fragmented assemblies, database bias, and genotype–phenotype discordance can cause false-positive or false-negative interpretation.	Low to moderate; generally scalable for large isolate collections and suitable for automated safety-screening pipelines.	Use as a safety-gate component. Report database version, identity and coverage thresholds, gene completeness, intrinsic or acquired status, genomic context, and phenotypic MIC validation where relevant.
Virulence and toxin screening	VFDB [18], VFanalyzer [18], Victors [163], species-specific toxin databases	Genome assembly or proteins	Virulence-associated and toxin-gene matches	Identifies safety-relevant loci, especially in taxa with opportunistic or pathogenic relatives.	Virulence databases may contain broad colonization, fitness, or low-specificity genes that require taxon-aware interpretation.	Low to moderate; computationally scalable, but manual curation of ambiguous hits can become time-intensive.	Use for safety-risk triage. Report confidence criteria, gene completeness, coding integrity, genomic context, taxonomic relevance, and whether hits were manually curated.
Plasmid detection and mobility context	PlasmidFinder [58], MOB-suite [57], plasmidSPAdes [147]	Genome assembly	Plasmid replicons; plasmid contigs; mobility predictions	Helps determine whether AMR, virulence, or functional genes may be plasmid-associated or transferable.	Short-read assemblies may misassign plasmid localization or fail to resolve complete plasmid structure.	Moderate; demand depends on assembly size and plasmid-reconstruction method. Large collections require batch execution and careful storage management.	Use when transferability or gene localization is important. Report whether plasmids are complete and circular, whether target genes are plasmid-linked, and whether long-read evidence supports localization.
Prophage and viral-region prediction	PHASTER [21], PhiSpy [164], geNomad [15]	Genome assembly	Predicted prophage or viral regions	Helps interpret genome stability, accessory genes, and potential cargo near safety or functional loci.	Predicted prophage boundaries, completeness, and activity may be uncertain, especially in fragmented assemblies. Prophage predictions can vary across tools and may represent intact, incomplete, degraded, cryptic, or uncertain regions. Prediction alone does not prove excision, induction, infectivity, cargo expression, or mobility.	Moderate; scalable across isolate genomes, but runtime and manual review increase with genome number and prophage complexity. Report confidence and completeness, compare tools when needed, and inspect genomic context before inferring activity, cargo relevance, or mobility.	Use to contextualize genome stability and accessory loci. Report prophage completeness, contig context, prediction category, and whether prophage predictions overlap key genes.
Insertion sequences and MGEs	ISEScan [20], MobileElementFinder [19], ISfinder [165]	Genome assembly	IS elements, transposons, mobile-element annotations	Supports interpretation of gene mobility, genome plasticity, and transfer risk.	Fragmented contigs may obscure complete mobile-element structure and linkage to target genes.	Low to moderate; scalable for isolate genomes, although mobile-context interpretation may require manual inspection.	Use to assess mobility context. Report element type, location relative to AMR, virulence, toxin, or functional genes, and contig completeness.
Secondary metabolite and bacteriocin mining	antiSMASH [24], BAGEL4 [25], DeepRiPP [166], RiPPMiner [167]	Genome assembly or predicted proteins	Predicted biosynthetic gene clusters; bacteriocin and RiPP predictions	Nominates antimicrobial potential, bacteriocin loci, and specialized metabolite clusters for validation.	Cluster presence does not confirm expression, product structure, secretion, immunity, antimicrobial activity, or ecological relevance.	Moderate; scalable for isolate genomes, but large genome collections may require HPC or batch workflows.	Use for functional hypothesis generation. Report cluster completeness, structural genes, immunity genes, transport genes, similarity to known clusters, and antimicrobial assay plan.
CAZyme and prebiotic-utilization analysis	CAZy [22], dbCAN [55], eggNOG-mapper [27]	Predicted proteins	CAZyme families; glycosidases; carbohydrate-utilization modules	Predicts carbohydrate metabolism, glycan adaptation, and possible prebiotic utilization.	CAZyme family assignment does not prove substrate specificity, pathway completeness, transporter availability, or growth on a substrate.	Moderate; scalable for predicted protein sets, but substrate interpretation and pathway review often require manual curation.	Use for carbohydrate-use hypotheses. Report CAZyme families, substrate interpretation limits, pathway completeness, transporters where relevant, and growth or substrate-validation assays.
Functional trait pathway reconstruction	KEGG [142], MetaCyc [143], eggNOG-mapper [27], manual curation	Genome annotation	Putative pathways for GABA, vitamins, EPS, stress response, BSH, and metabolism	Converts gene lists into pathway-level, testable biological hypotheses.	Incomplete pathways, missing transporters, missing regulators, or precursor dependence can limit biological activity.	Moderate; automated annotation is scalable, but pathway-completeness review and biological interpretation are manual and time-intensive.	Use to move beyond single-gene interpretation. Report tool and version, database release and accession date where applicable, pathway completeness, missing genes, gene context, and planned validation assay.
Pangenome analysis	Roary [48], Panaroo [45], PIRATE [168], PPanGGOLiN [169]	Annotated genomes	Core and accessory genome matrix; gene presence or absence table	Identifies conserved, accessory, lineage-associated, or strain-specific traits across comparator genomes.	Results depend on genome selection, annotation consistency, clustering thresholds, and species or genus breadth.	High; memory and CPU demand increase rapidly with genome number, annotation size, and clustering strategy. Large datasets usually require HPC.	Use for comparative and evolutionary interpretation. Report genome set selection, annotation consistency, clustering threshold, filtering criteria, and core or accessory definitions.
Genomic distance and rapid comparison	Mash [46], FastANI [3], sourmash [170]	Genome assemblies	Distance matrix; similarity estimates	Supports large-scale comparator selection, dereplication, species confirmation, and rapid population context.	Approximate distances may require confirmation by ANI, phylogenomics, or type-strain comparison for formal taxonomic interpretation.	Low to moderate; highly scalable and suitable for large genome collections, dereplication, and rapid comparator selection.	Use for screening and comparator selection. Report sketch size, k-mer size, thresholds, and comparator-genome inclusion criteria.
Phylogenomics	IQ-TREE [47], RAxML-NG [171], FastTree [172], PhyloPhlAn [173]	Core genes, SNPs, or marker-gene alignments	Phylogenomic tree with support values	Places candidate strains in evolutionary context and supports trait-overlay interpretation.	Tree topology can be affected by recombination, poor alignment, uneven sampling, model choice, and low-quality genomes.	Moderate to high; demand depends on genome number, alignment length, model complexity, and bootstrap strategy. Large analyses usually require HPC.	Use for evolutionary context and lineage-aware interpretation. Report alignment method, model selection, bootstrap or support method, rooting strategy, recombination handling where relevant, and taxa included.
Trait visualization and decision outputs	R, ggplot2, ComplexHeatmap, Python, iTOL [174], custom workflow tables	Matrices from safety, functional, and comparative analyses	Heatmaps, decision matrices, trait overlays, prioritization tables	Converts complex outputs into auditable strain-ranking, safety-gated prioritization, and validation-planning evidence.	Visual summaries can oversimplify uncertainty if thresholds, missing data, or decision rules are not transparent.	Low to moderate; scalable for prepared matrices, but very large heatmaps or interactive dashboards may require optimized scripts and sufficient memory.	Use for reporting and decision support. Report code availability, input tables, thresholds, missing-data handling, and decision rules.

## Safety-Gated Probiogenomics

Safety-gated interpretation distinguishes probiogenomics from simple genome annotation by converting safety-relevant findings into structured early-stage risk-triage decisions. In this review, the safety gate should not be treated as a deterministic pass or fail system; instead, findings should be interpreted across 5 evidence levels: genomic detection, biological plausibility, potential mobility, demonstrated mobility, and demonstrated phenotypic activity. Genomic detection refers to the presence of a database hit or predicted locus. Biological plausibility considers gene completeness, coding integrity, domain structure, taxonomic relevance, genomic neighborhood, and whether the locus is likely to be functional. Moreover, potential mobility indicates association with a plasmid, prophage, transposon, integrative element, or other MGE, while demonstrated mobility requires stronger evidence such as complete mobile-element reconstruction, mobilization or conjugation evidence, host-range information, or experimental transfer data. Demonstrated phenotypic activity requires matched laboratory evidence, such as minimum inhibitory concentration (MIC) testing, hemolysis or toxin assays, metabolite quantification, or other targeted safety assays [7,8,69]. A candidate with attractive predicted functions should not advance if its identity is uncertain, genome quality is inadequate, or unresolved safety signals remain. The safety gate should use predefined evidence categories, including taxonomic confidence, genome quality, gene identity and coverage, gene completeness, intrinsic versus acquired status, chromosomal versus mobile context, phenotype support, intended-use relevance, and regulatory context. Evidence should be interpreted by confidence, consequence, and intended use rather than by annotation presence alone. For example, a complete acquired AMR gene with mobility and phenotype support has stronger safety implications than a low-identity partial hit on a fragmented contig. Similarly, a complete toxin locus with plausible genomic context should be interpreted differently from a broad, low-specificity virulence annotation. Negative database searches should be reported as no high-confidence risk signal detected under specified tools, databases, thresholds, and genome quality, rather than as proof of safety [13,16,18,56].

**Fig. 3.**
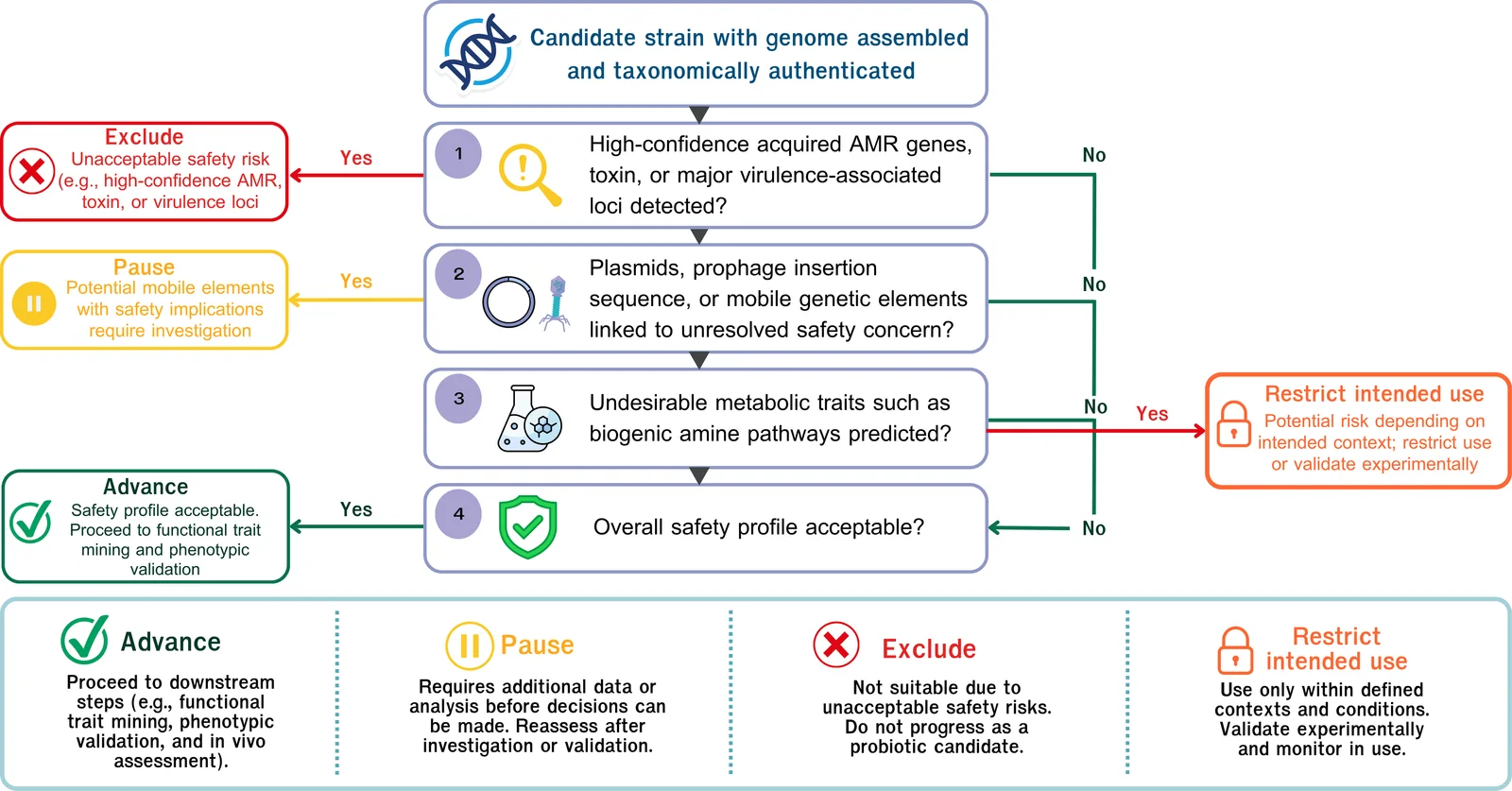
Genome-based safety decision tree for candidate probiotic prioritization. Safety-relevant genomic findings should be interpreted as decision-support evidence. Depending on the confidence and context of AMR, virulence, toxin, mobile-element, or undesirable-metabolism signals, a strain may advance, pause for further investigation, be excluded, or be restricted to a defined intended use.

Safety interpretation is also organism- and application-dependent. Lactobacillaceae, *Bifidobacterium*, *Pediococcus*, *Weissella*, *Bacillus*, and *Enterococcus* candidates raise different safety questions because their histories of use, opportunistic potential, intrinsic resistance, mobile-element burden, and intended applications differ. Thus, safety should be interpreted in relation to the intended host, population vulnerability, route of exposure, dose, product matrix, manufacturing process, phenotypic safety evidence, and jurisdiction-specific requirements [70]. The decision categories proposed here are operational criteria for early-stage prioritization, not universal regulatory cutoffs. Uncertainty should remain visible when safety calls depend on borderline identity or coverage, unresolved mobile context, conflicting genotype–phenotype evidence, or taxon-specific interpretation [2,5,6]. This decision-oriented interpretation is summarized in Fig. 3 and Table 3, where safety-relevant findings are translated into advance, pause, exclude, or restrict-intended-use outcomes. These decisions support prioritization and validation planning, but do not replace phenotypic safety testing or regulatory assessment.

**Table 3.** Safety-gated genomic screening framework. The safety gate is an early-stage risk-triage and prioritization framework, not a deterministic safety certification system. Findings should be interpreted across 5 evidence levels: genomic detection, biological plausibility, potential mobility, demonstrated mobility, and demonstrated phenotypic activity. Regulatory and intended-use requirements are summarized in Table 4.

Safety domain	What to screen	Key interpretive question	Example tools/resources
Taxonomic reliability	Species and strain identity, mislabeling, mixed-culture concerns or contamination concerns	Is the candidate truly what it is claimed to be?	FastANI, GTDB-Tk, phylogenomics
AMR genes and mutations	Acquired AMR genes, resistance-associated mutations intrinsic *vs*. acquired resistance, gene completeness, and mobility context	Is there evidence of transferable or clinically important resistance?	AMRFinderPlus, CARD or RGI, ResFinder
Virulence- and toxin-associated genes	Toxins, cytolysin-like regions, pathogenicity-associated loci, low-specificity virulence hits, and mobile-context-associated virulence regions	Are there high-risk virulence signals that would limit use?	VFDB, taxon-specific databases where available, manual curation
Plasmids and MGEs	Replicons, transposases, insertion sequences, integrative elements, transposons, integrons, and MGE-adjacent safety genes	Could predicted traits be transferable or unstable, or horizontally acquired?	PlasmidFinder, MobileElementFinder, ISEScan
Prophages and viral regions	Intact, incomplete, or questionable prophage regions	Could viral regions affect genome stability, transfer, or cargo interpretation?	PHASTER, geNomad
Undesirable metabolites	Biogenic amine pathways, other unwanted food-associated traits, pathway completeness, precursor availability, and metabolite accumulation	Could the strain generate undesirable products under intended conditions?	Annotation + pathway curation
Hemolysis and overt phenotype risk	Hemolysis-associated traits, adverse phenotype screens, genotype–phenotype contradictions	Does the phenotype support or contradict genomic reassurance?	Blood agar assay, targeted phenotypic testing
Regulatory and intended-use context	Proposed use as human probiotic, animal feed probiotic, aquaculture probiotic, plant-associated beneficial microbe, starter or fermentation culture, dietary supplement, or live biotherapeutic product; target host or population; route of exposure; dose; product matrix; manufacturing process; environmental-release potential; jurisdiction; and regulatory pathway	Does the genomic and phenotypic evidence support the proposed use and claim level?	EFSA QPS, FDA GRAS, FDA live biotherapeutic product guidance, FAO/WHO probiotic evaluation principles, national guidance
Overall safety gate	Integration of all safety-relevant evidence, intended-use context, and regulatory relevance	Should the strain advance, pause, be excluded, or be restricted to specific uses?	Structured decision table

### Antimicrobial resistance

AMR screening is one of the most important safety modules because candidate probiotic strains and other live microbial candidates may persist in microbial communities or enter food, host-associated, environmental, or production ecosystems. AMR results should be separated into intrinsic, acquired, ambiguous, and phenotype-supported categories. Intrinsic, chromosomal, nontransferable resistance has different implications from an acquired determinant linked to plasmid, transposon, or insertion-sequence markers. Thus, AMR interpretation should prioritize gene completeness, identity, coverage, contig context, and mobility rather than simple gene counts [13,17,19]. AMR reporting should distinguish regulatory-context-specific requirements from broader best practice. For microorganisms intentionally used in the food chain or evaluated in EFSA-related contexts, WGS-based AMR reporting should align with EFSA recommendations, including use of at least 2 maintained AMR databases and reporting of software, parameters, database version or access date, subject sequence accession, predicted function, percentage identity, and percentage coverage. In this regulatory context, hits with at least 80% identity and 70% subject-sequence coverage should be reported; fragmented hits with at least 80% identity to the same AMR gene should also be checked for full-gene recovery [69]. In other probiogenomic contexts, such as early discovery, live biotherapeutic development, plant-associated microbes, aquaculture strains, or research isolates, these criteria are best interpreted as useful reporting guidance rather than universal regulatory requirements. EFSA recommendations therefore provide a regulatory-oriented reference for specific food-chain applications, whereas genome-quality assessment, database transparency, identity and coverage reporting, and genotype–phenotype correlation represent broader methodological best practices applicable across probiogenomic studies.

For safety-gated prioritization, AMR findings should be interpreted by whether the determinant is intrinsic or acquired, complete or truncated, high- or low-confidence, chromosomal or mobile-element-associated, and concordant or discordant with phenotypic susceptibility testing [71]. AMRFinderPlus, CARD or RGI, ResFinder-type tools, and related resources formalize AMR detection, but they do not remove the need for interpretation. A low-identity partial hit, an intrinsic marker, and a complete acquired AMR gene on a plasmid-associated contig should not be treated as equivalent. Similarly, an acquired AMR gene near a plasmid replicon or mobility marker should be reported as plasmid-associated or potentially mobile only when supported by genomic-context evidence; demonstrated transferability requires stronger evidence such as plasmid reconstruction, mobilization or conjugation markers, host-range information, or experimental transfer data.

Borderline or fragmented hits should be reported as ambiguous findings requiring manual review rather than treated automatically as exclusionary. False-positive AMR calls may arise from partial genes, low-specificity matches, conserved domains, pseudogenes, contamination, mixed cultures, or permissive thresholds [72]. False-negative results may occur when resistance is caused by divergent genes, regulatory changes, target-site mutations, efflux regulation, copy-number variation, expression differences, or assembly fragmentation [73]. Therefore, genome quality, read depth, contamination, assembly fragmentation, database choice, and manual curation should be considered before AMR results are used for candidate exclusion or advancement. However, phenotypic antimicrobial susceptibility testing remains necessary when safety decisions depend on antimicrobial susceptibility. Genotype may fail to predict phenotype, and a detected gene may be incomplete, silent, or not associated with measurable resistance under tested conditions. When genotype and phenotype conflict, the safety gate should use the more conservative interpretation until additional evidence resolves the discrepancy [74,75]. Elevated MIC values without a detected AMR gene should prompt investigation of intrinsic resistance, mutations, regulatory mechanisms, or database limitations, whereas a detected acquired AMR gene without phenotypic resistance still requires review of gene completeness, expression potential, and mobility context [76]. Thus, genome screening should guide phenotype testing and interpretation but should not replace MIC testing when AMR safety decisions are required [69,74–76].

### Virulence-associated genes and toxins

Virulence screening should be framed as risk triage rather than pathogen diagnosis. Many commensal or food-associated bacteria encode surface, stress-response, iron-acquisition, adhesion, or transporter genes that resemble entries in virulence databases. A similarity hit therefore does not automatically prove virulence. False-positive virulence calls may arise when broad or low-specificity database entries annotate context-dependent colonization, adhesion, stress-response, transport, or iron-acquisition genes as virulence-associated [77]. Virulence databases may also be biased toward clinically important pathogens, so annotations derived from distant taxa should be interpreted cautiously in candidate probiotic backgrounds.

For safety-gated prioritization, virulence and toxin findings should be interpreted according to evidence strength, taxonomic relevance, gene completeness, genomic context, mobility, and intended use. Low-specificity annotations should be labeled as ambiguous, whereas high-confidence toxin genes, complete cytolysin-like loci, pathogenicity-island components, or virulence regions linked to mobile elements should trigger conservative interpretation [18,78,79]. A partial similarity hit to a broad adhesion- or stress-associated protein may warrant manual review, whereas a complete toxin locus or high-confidence virulence region in a pathogenicity island, plasmid-like contig, prophage, or other mobile context may support a pause, exclusion, or restricted-use decision, depending on evidence confidence, intended use, and regulatory context [69,80].

The most useful virulence report describes the exact gene or region, identity and coverage, completeness, genomic neighborhood, taxonomic background, and whether the locus is plausibly functional. Functional plausibility should be assessed using gene completeness, coding-sequence integrity, premature stop codons or frameshifts, conserved domain architecture, locus organization, neighboring genes, and association with pathogenicity islands, plasmids, prophages, or other mobile elements. Domain-based annotation resources, taxon-specific reference genomes, comparative genomic context, manual curation, and targeted phenotypic safety tests can help distinguish complete and contextually plausible risk loci from low-specificity database matches [81].

False-negative virulence screening is also possible when toxin or virulence genes are absent from the database, highly divergent, fragmented by assembly breaks, missed by gene prediction, or regulated by variants not captured by simple gene-presence analysis. For genera with opportunistic members, such as *Enterococcus* or *Bacillus*, virulence screening should be interpreted together with taxonomic placement, source, intended use, and phenotypic safety tests [32,35,41]. When genomic predictions and phenotypic observations conflict, the safety gate should again use a conservative interpretation until the discrepancy is resolved.

### Plasmids, prophages, and MGEs

MGEs are central to probiogenomic safety because they connect gene content with genomic context, potential mobility, stability, and strain specificity. Plasmids, prophages, insertion sequences, integrative elements, and transposons may carry AMR genes, toxin or virulence-associated loci, bacteriocin clusters, stress-response systems, carbohydrate-utilization loci, or other accessory traits. The same accessory feature that makes a strain functionally distinctive may also make the trait unstable, potentially mobile, or recently acquired. Thus, mobile elements should be interpreted not only as safety concerns but also as evolutionary drivers of niche adaptation, functional diversification, and strain-level variation [14,15,19,20]. Mobile-context interpretation should consider whether a target gene is chromosomal, plasmid-associated, prophage-associated, MGE-adjacent, potentially mobile, experimentally transferable, or unresolved because of assembly fragmentation. These categories are not equivalent. Plasmid detection should be distinguished from plasmid reconstruction: PlasmidFinder-type results mainly support replicon detection or typing, whereas MOB-suite-type analyses can provide additional reconstruction and mobility-related characterization. However, detecting a plasmid replicon in the same assembly as an AMR gene, virulence-associated locus, or functional trait does not prove that the gene is physically located on that plasmid unless supported by contig linkage, complete plasmid reconstruction, read-depth patterns, circularization evidence, or long-read or hybrid assembly. Similarly, prophage predictions should not be treated as binary present-or-absent calls. Predicted prophage regions should be classified according to confidence and completeness, such as intact, questionable, incomplete, degraded, cryptic, or tool-discordant, because different prediction methods may identify different boundaries or genomic regions. Prediction alone does not demonstrate excision, induction, infectivity, cargo expression, or biological mobility; therefore, prophage-associated genes should be interpreted using prediction confidence, genomic context, cargo content, and supporting experimental evidence where available. However, mobile-element-associated does not mean demonstrated transferable. A plasmid-like contig does not prove plasmid structure, proximity to an insertion sequence or transposase does not prove mobilization, and prophage association does not prove excision, induction, infectivity, cargo expression, or horizontal transfer [82,83].

Mobile-context calls are vulnerable to false-positive and false-negative interpretation because fragmented assemblies, collapsed repeats, uncertain prophage boundaries, and marker-limited predictions can misassign AMR, virulence, toxin, or functional genes to plasmid, prophage, or MGE contexts. Therefore, potentially mobile findings should require supporting evidence such as contig linkage, read-depth patterns, circularization, mobility genes, integrase or transposase context, plasmid or prophage reconstruction, tool agreement, or long-read or hybrid confirmation. Evidence for experimentally transferable traits requires direct experimental or epidemiological support, such as conjugation, mobilization, transformation, host-range testing, or demonstrated dissemination. Unresolved mobility should usually trigger a pause decision when the region contains AMR genes, toxin genes, high-confidence virulence-associated loci, or undesirable metabolic pathways. Conversely, a functional trait located in a stable chromosomal region and not linked to mobility markers may support advancement to validation if other safety evidence is acceptable. Candidate reports should therefore state localization confidence and supporting evidence, including contig length, coverage pattern, circularization status, nearby mobility genes, plasmid or prophage prediction confidence, and whether long-read or hybrid assembly supports the inferred context [42,43,84,85].

### Biogenic amines and undesirable metabolic traits

Functional sections of probiotic papers often emphasize beneficial metabolites, but safety assessment must also ask whether a strain can produce undesirable compounds. In food-associated bacteria, genes involved in histidine-to-histamine, tyrosine-to-tyramine, ornithine-to-putrescine, lysine-to-cadaverine, agmatine-to-putrescine, or other biogenic amine pathways may be relevant when amino acid substrates and permissive fermentation conditions are present [86,87]. Gene presence does not prove harmful accumulation, but it identifies a testable risk. Undesirable-metabolism findings should be interpreted according to pathway completeness, transporter presence, precursor availability, regulatory context, product matrix, pH, salt concentration, fermentation temperature, storage conditions, growth phase, gene expression, and quantitative metabolite evidence. A decarboxylase annotation should therefore lead to targeted validation rather than automatic exclusion or reassurance because a single gene does not necessarily indicate an intact pathway, active expression, sufficient substrate availability, or biologically relevant amine accumulation [33]. Matrix-aware assays such as high-performance liquid chromatography (HPLC) or liquid chromatography–mass spectrometry (LC-MS) are needed to determine whether compounds accumulate under intended fermentation, storage, or host-associated conditions. A complete pathway with measurable accumulation under intended-use conditions may justify exclusion or restriction, whereas an incomplete pathway, isolated gene fragment, or low-confidence annotation should usually trigger manual review or targeted testing. Thus, biogenic amine risk should be reported as an evidence-based interpretation integrating genome prediction, pathway completeness, environmental conditions, and direct metabolite quantification, not as a simple gene-present or gene-absent conclusion.

### Safety-gated prioritization

A safety-gated model can translate genome screening into 4 practical decisions: advance, pause, exclude, or restrict intended use. These decisions are early-stage risk-triage outcomes rather than final safety or regulatory conclusions. They should follow the strength, confidence, and consequence of evidence. A strain may advance when identity, genome quality, safety screens, intended-use context, and basic phenotypic evidence are coherent, with no unresolved high-confidence safety concern. A strain should pause when evidence is incomplete or ambiguous, including ambiguous hits, assembly limitations, unresolved mobile context, partial genes, low-specificity database matches, conflicting database predictions, genotype–phenotype conflict, regulatory uncertainty, or insufficient intended-use evidence prevents confident interpretation. Exclusion is justified when unresolved high-confidence AMR, toxin, virulence, or transferable safety concerns remain incompatible with the proposed use, especially when supported by gene completeness, biological plausibility, mobility evidence, and/or phenotype. Restriction may be appropriate when risk is specific to a host group, product matrix, dose, route of exposure, manufacturing process, regulatory pathway, or intended application [5,6].

The safety-gated probiogenomic framework is modular rather than one-size-fits-all. Human probiotics, animal feed probiotics, aquaculture probiotics, plant-associated beneficial microbes, starter cultures, dietary supplements, and live biotherapeutic products require different safety questions, validation endpoints, exposure assumptions, environmental-release considerations, and regulatory evidence [88]. Accordingly, human candidates may require host-response and population-specific safety evidence, animal or aquaculture candidates may require food-chain or environmental-risk assessment, plant-associated strains may require ecological-impact evaluation, and fermentation strains may prioritize product quality, metabolite safety, and manufacturing consistency [89,90].

Decision logic should be based on predefined evidence criteria, including gene identity and coverage, gene completeness, intrinsic versus acquired status, chromosomal versus mobile context, taxonomic relevance, phenotype support, genotype–phenotype agreement, intended-use context, and regulatory expectations. This logic should be reported transparently so readers can understand why a candidate was advanced, paused, excluded, or restricted. Such noncompensatory interpretation prevents attractive predicted functions from overriding unresolved safety concerns. As summarized in Table 4, final interpretation should also consider intended use, host population, product matrix, manufacturing context, phenotypic safety evidence, and jurisdiction-specific requirements. Importantly, safety-gated probiogenomic decisions should not be interpreted as regulatory determinations. QPS, GRAS, and live biotherapeutic product (LBP) pathways require separate evaluation according to their specific regulatory frameworks, intended use, manufacturing requirements, and supporting evidence.

**Table 4.** Regulatory and intended-use contexts for probiogenomic interpretation

Regulatory or intended-use context	Typical application	Main evidence requirements	Key safety considerations	Role of probiogenomics
Candidate probiotic	Discovery-stage strains intended for future food, feed, supplement, clinical, or environmental applications	Strain identity, genome quality, safety screening, functional prediction, intended-use definition, and matched validation	AMR, virulence, toxins, MGEs, undesirable metabolites, taxonomy, and genome quality	Supports early prioritization and validation planning, but does not establish probiotic status
Food or dietary probiotic product	Foods, fermented products, capsules, powders, or dietary supplements	Strain identity, product quality, dose, safety evidence, and substantiated benefit for the claimed use	Product matrix, target population, dose, manufacturing consistency, AMR, virulence, toxins, and undesirable metabolites	Supports strain authentication, risk screening, and functional hypothesis generation
EFSA QPS-related food or feed microorganisms	Microorganisms intentionally added to food or feed in the European context	Taxonomic identity, QPS status of the taxonomic unit, relevant QPS qualifications, strain-level assessment where required, and evidence that the proposed use fits the assessed taxonomic unit and qualification	Acquired AMR, toxigenicity, pathogenicity, intended use, qualification-specific safety concerns, and strain-specific findings not resolved by taxonomic-level QPS status	Helps confirm taxonomy and detect strain-level genomic risk signals, but QPS is a taxonomic-unit pre-assessment framework and does not by itself constitute strain-level market authorization or proof of probiotic safety
FDA GRAS-related food use	Microorganisms or microbial-derived substances intended for food use in the United States	Intended-use definition, safety evidence, exposure assessment, manufacturing information, specifications, and expert evaluation supporting a GRAS conclusion under the conditions of intended use	Strain identity, history of use, toxigenicity, AMR, exposure level, product consistency, manufacturing process, specifications, and intended-use conditions	Provides genome-based support for identity and risk assessment, but a GRAS conclusion depends on the totality of generally available safety evidence for a defined use and should not be described as generic regulatory approval
Live biotherapeutic product	Live microorganisms developed for prevention, treatment, or mitigation of disease	Defined strain identity, manufacturing control, dose, stability, target-population safety, clinical evidence, and regulatory review within a drug or biologic development pathway	Clinical safety, vulnerable populations, persistence, shedding, AMR, virulence, genetic stability, and product consistency, purity, potency, quality control, and manufacturing comparability	Supports strain definition, genomic safety assessment, stability monitoring, and mechanism-oriented validation, but clinical acceptability requires product-specific manufacturing, nonclinical, clinical, and regulatory evidence beyond genome screening
Animal feed probiotic	Feed additives or microbial products for livestock, poultry, companion animals, or other animals	Host-specific safety, feed stability, dose, performance or health endpoints, and food-chain relevance where applicable	AMR transfer risk, animal safety, environmental exposure, food-chain safety, and manufacturing consistency	Helps assess taxonomy, AMR or virulence risk, MGEs, and host-specific validation priorities
Aquaculture probiotic	Microbial products used in aquatic animals or aquaculture systems	Aquatic host safety, pathogen-challenge data, water-system compatibility, persistence, and environmental assessment	Environmental release, horizontal gene transfer, persistence in water systems, pathogen interaction, and food-chain relevance	Supports safety screening, ecological-risk assessment, strain tracking, and functional prioritization
Plant-associated beneficial microbe	Bioinoculants, plant-growth-promoting strains, or rhizosphere or phyllosphere applications	Plant compatibility, colonization, performance endpoints, environmental release, and ecological impact	Environmental persistence, AMR or virulence-like loci, nontarget effects, and ecosystem compatibility	Supports colonization-trait prediction, ecological-risk screening, and validation planning
Starter or fermentation culture	Food fermentation, product development, texture, flavor, preservation, or process improvement	Matrix-specific growth, product consistency, metabolite profile, manufacturing stability, and food safety	Biogenic amines, undesirable metabolites, toxins, AMR, phage sensitivity, and product-matrix behavior	Supports strain authentication, metabolite-risk screening, pathway prediction, and matrix-specific validation
Postbiotic or nonliving microbial product	Inactivated cells, cell components, or microbial metabolites	Product composition, manufacturing control, safety, bioactivity evidence, and intended-use definition	Residual toxins, undesirable metabolites, inflammatory components, allergens, and product consistency	Helps characterize the source strain and biosynthetic potential, but final evidence depends on product composition and bioactivity testing

- Advance: coherent identity, acceptable genome quality, no unresolved high-confidence safety signal, and suitable intended-use context.

- Pause: ambiguous hit, borderline taxonomy, insufficient assembly quality, partial or low-specificity gene match, unresolved plasmid or prophage or MGE context, conflicting database predictions, or genotype–phenotype conflict requiring additional data.

- Exclude: unresolved high-confidence AMR, toxin, virulence, or transferable safety concern incompatible with the proposed use, especially when supported by complete loci, biological plausibility, mobility evidence, or phenotype.

- Restrict intended use: context-dependent safety profile requiring limited application, additional validation, specific host or product conditions, or regulatory constraints.

Regulatory interpretation should be treated as an intended-use-specific evidence layer rather than as a direct output of genome screening [91,92]. To reduce repetition across the manuscript, Table 4 summarizes major regulatory and intended-use contexts relevant to probiogenomic interpretation.

## Functional Trait Mining

Functional trait mining begins after unsuitable candidates have been removed or paused by the safety gate. Its purpose is not to prove probiotic efficacy, but to identify biologically plausible traits that justify targeted validation. Because a genome contains thousands of annotations, functional mining should be selective and guided by the intended application, delivery route, product matrix, host site, and proposed mechanism. Oral candidates may prioritize gastrointestinal survival, acid and bile tolerance, stress response, adhesion, and compatible substrate use, whereas fermentation or synbiotic candidates may require deeper analysis of carbohydrate metabolism, EPS, antimicrobial compounds, or metabolite production [39,50]. However, these traits should be described as genome-predicted functional features relevant to candidate evaluation, not as inherently beneficial probiotic properties. Adhesion, bile salt hydrolase (BSH) activity, EPS production, oxidative-stress tolerance, antimicrobial activity, carbohydrate utilization, and immunomodulatory potential can be favorable, neutral, or undesirable depending on host context, dose, product matrix, ecological setting, mechanism, and intended claim. Functional interpretation should move beyond isolated gene presence toward pathway- and systems-level evidence. Tools based on alignment, hidden Markov model (HMM) profiles, orthology, domain annotation, or rule-based BGC detection can nominate candidate genes and loci, but claim-critical predictions should also consider gene completeness, coding integrity, genomic neighborhood, regulatory context, pathway completeness, taxonomic relevance, comparative distribution, and manual curation. False functional predictions may arise from fragmented loci, pseudogenes, missing transporters or regulators, incomplete pathways, poor expression, or absence of the required substrate, cofactor, pH, matrix, or host condition. Therefore, functional calls should be used to generate hypotheses and select validation assays rather than to establish efficacy [33,93–96].

**Fig. 4.**
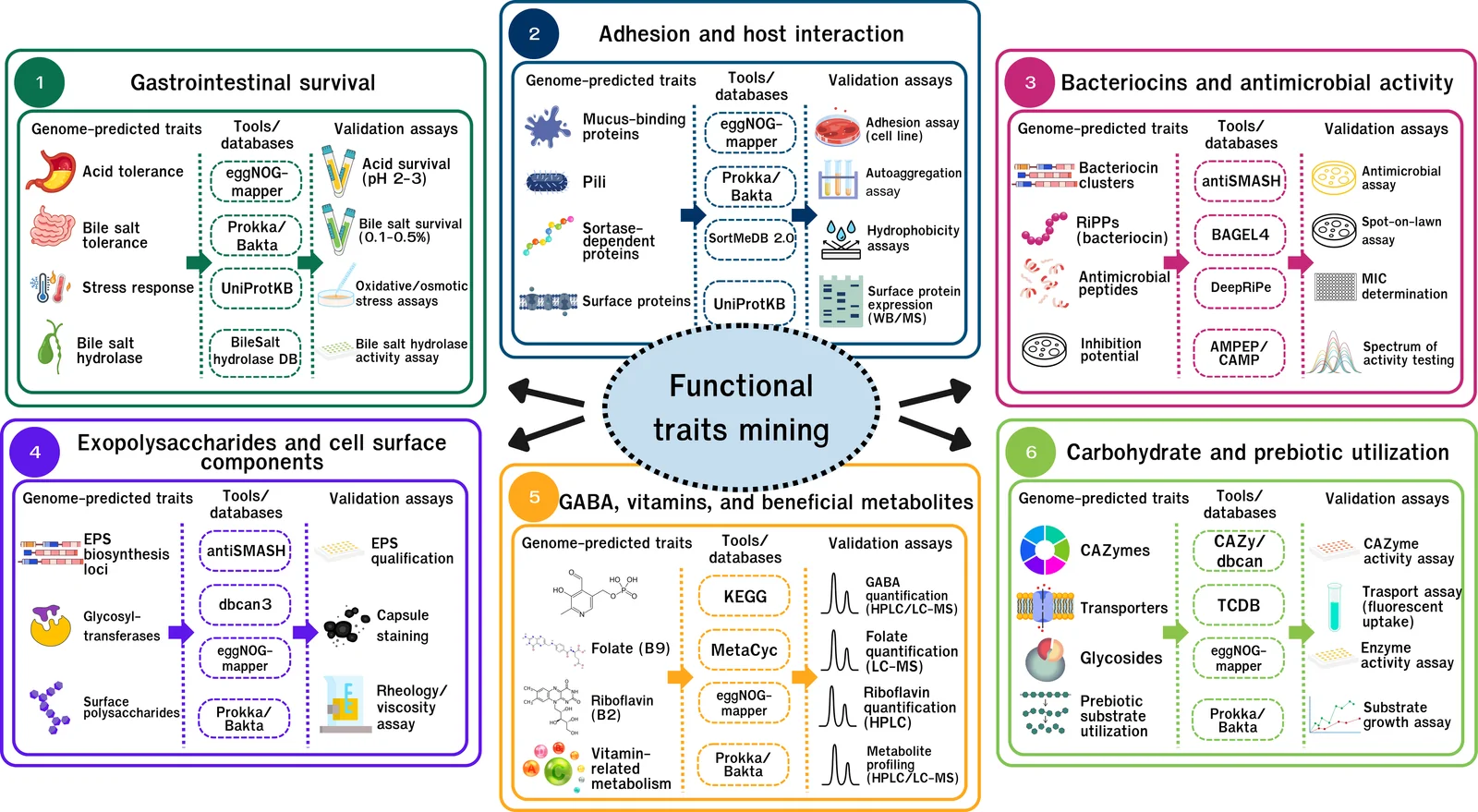
Functional trait mining in probiogenomics. Genome-predicted traits are grouped into major functional domains, including gastrointestinal survival, host interaction, antimicrobial potential, EPS and surface architecture, beneficial metabolites, and carbohydrate and prebiotic utilization. These predictions should be interpreted at pathway and systems levels and linked to matched validation assays before phenotype-level claims are made.

A useful functional analysis links each predicted locus or pathway to its biological rationale and matched assay. For example, BSH annotations require bile-response or BSH activity assays; mucus-binding or pili-associated genes require adhesion or host-response models; CAZyme profiles require substrate-growth assays; bacteriocin clusters require antimicrobial assays with acid, peroxide, and protease controls; EPS loci require polymer extraction and characterization; and metabolite-related pathways require targeted quantification. Transcriptomics, proteomics, and metabolomics can further test whether predicted systems are expressed, translated, and biochemically active under relevant conditions. Evidence should be reported with claim-matched language, such as genome-predicted, putative, condition-dependent, or validated under defined conditions, rather than implying broad probiotic benefit from gene presence alone. Functional novelty should also be interpreted cautiously when traits are accessory, lineage-restricted, or mobile-element-associated, because such features may be unstable, transferable, or niche-specific. Figure 4 and Table 5 summarize the main functional domains and matched validation assays.

**Table 5.** Genome-predicted functional traits relevant to candidate probiotic evaluation, representative genomic markers, and validation assays

Functional domain	Representative genomic markers or pathways	Example tools	Recommended validation assays
Acid and bile tolerance	Stress-response genes, chaperones, membrane remodeling, bile salt hydrolase	Prokka or Bakta + manual curation	Simulated gastric survival, bile tolerance, BSH activity assay
Adhesion or host interaction	Mucus-binding proteins, pili, sortase-dependent proteins, S-layer proteins, surface polysaccharides	Annotation + comparative genomics	Mucin-binding assays, Caco-2/HT-29 adhesion, host-response assays
Antimicrobial potential	Bacteriocin and RiPP clusters, transporters, immunity proteins	BAGEL4, antiSMASH	Agar diffusion with acid/peroxide controls, purification, expression support
EPS or cell-surface architecture	Glycosyltransferases, polymerization or export loci, precursor pathways	General annotation + locus inspection	EPS extraction, quantification, structural characterization, rheological and functional assays
GABA production	*gadB*, *gadC* and related glutamate decarboxylase modules	Annotation + pathway curation	HPLC/LC-MS quantification of GABA under defined culture conditions
Vitamin production	Folate, riboflavin, B-group vitamin biosynthetic pathways	eggNOG-mapper, pathway annotation	Targeted metabolite quantification
Carbohydrate or prebiotic utilization	CAZymes, transporters, substrate-specific catabolic pathways	CAZy, dbCAN	Growth on defined substrates, substrate disappearance, metabolite production
Oxidative stress response	Catalases or peroxidases where relevant, thioredoxin or glutaredoxin systems, chaperones	Annotation	Oxidative stress survival assays
Immunomodulatory potential	Surface molecules, EPS, metabolites, peptidoglycan-associated features	Annotation + comparative inference	Cell-based immune assays, cytokine profiling and barrier-function assays where relevant

### Gastrointestinal survival

Gastrointestinal survival reflects multiple stresses, including low pH, bile salts, digestive enzymes, osmotic pressure, oxidative stress, nutrient limitation, and matrix-dependent protection. Genome analysis can identify candidate systems for stress response, membrane remodeling, chaperone activity, bile resistance, proton homeostasis, and oxidative defense, but survival should be tested under defined conditions [37,97,98]. Relevant assay parameters include matrix, pH, bile concentration, exposure time, growth phase, inoculum preparation, comparator strain, and viability endpoint. Limited genome-predicted stress capacity should not automatically exclude a strain, because lyophilization, encapsulation, protective carriers, or matrix-based delivery may improve stability and gastrointestinal survival; however, these strategies also require validation under relevant storage, processing, and gastrointestinal-simulation conditions.

### Adhesion and host interaction

Adhesion-associated predictions connect the bacterial genome to host or mucosal interfaces. Candidate features include mucus-binding proteins, sortase-dependent proteins, pili, S-layer proteins, surface polysaccharides, moonlighting proteins, and other cell-envelope components. Comparative genomics can determine whether these features are conserved or strain specific. For example, genome-resolved comparison of *Lactobacillus rhamnosus* GG nominated pili and mucus-binding proteins as plausible contributors to host interaction [34,99,100]. However, adhesion assays using mucin, Caco-2, HT-29, or related models demonstrate interaction only under test conditions and do not prove durable colonization or health benefit. These predictions should therefore guide adhesion or host-response assays rather than serve as direct evidence of probiotic efficacy [33].

### Bacteriocins and antimicrobial activity

Bacteriocin and RiPP (ribosomally synthesized and post-translationally modified peptide) mining is a clear example of genome-guided candidate selection. Specialized tools such as BAGEL4 and antiSMASH can identify candidate clusters, modification enzymes, transporters, immunity proteins, and regulatory components that may be missed by general annotation [24,25,101]. However, a predicted biosynthetic cluster does not guarantee expression, and an inhibition zone does not prove that the predicted bacteriocin caused the activity. Organic acids, hydrogen peroxide, nutrient depletion, or unrelated metabolites may produce similar effects. Stronger evidence requires neutralized supernatants, catalase or protease controls, spectrum testing, purification, expression analysis, or genetic confirmation. Until such evidence is available, the most defensible wording is that the genome encodes a putative antimicrobial cluster [102,103].

### EPS and cell surface components

EPS and other cell-surface polysaccharide structures, including capsular polysaccharides where relevant, may influence texture, viscosity, aggregation, drying tolerance, biofilm formation, adhesion, immune interaction, and stress resistance. Genome mining can identify glycosyltransferases, precursor pathways, polymerization and export systems, and regulatory genes, but a partial locus or isolated glycosyltransferase annotation is weak evidence [104–106]. EPS interpretation should match the proposed claim. Food-texture or fermentation-performance claims require rheological and fermentation assays; stress-tolerance claims require survival during drying, storage, or stress exposure; and host-interaction claims require adhesion or immune-response assays. Genome prediction alone cannot define polymer yield, monomer composition, linkage structure, or biological effect, so EPS claims should remain genome-predicted until extraction, quantification, structural characterization, and functional testing are performed [33].

### GABA, vitamins, and beneficial metabolites

Metabolite-oriented probiogenomics links genome content to measurable products. Pathways associated with GABA, folate, riboflavin, vitamin B12-related metabolism, organic acids, and other fermentation products can help prioritize strains for food, feed, or host-associated applications [103,104,107,108]. However, pathway completeness, transporter presence, precursor availability, cofactors, pH, matrix composition, growth phase, and regulation strongly affect metabolite accumulation. A strain may encode a pathway but fail to produce the compound under relevant conditions or produce an amount that is analytically detectable but biologically minor. Therefore, metabolite-oriented claims should be supported by pathway-completeness review and targeted quantification using HPLC, LC-MS, biochemical assays, or metabolomics under relevant substrate, matrix, growth, storage, and host-application conditions [33,109].

### Carbohydrate and prebiotic utilization

Carbohydrate utilization connects probiogenomics with diet, fermentation ecology, and synbiotic design. CAZymes, transporters, glycosidases, phosphotransferase systems, and catabolic pathways can suggest whether a candidate strain may use plant-derived fibers, oligosaccharides, milk glycans, or host-derived substrates. CAZy and dbCAN-type resources provide a foundation for annotation, while comparative genomics can determine whether substrate-utilization pathways are conserved, lineage-associated, or strain-specific [22,23,110,111]. Prebiotic compatibility should not be inferred from family-level enzyme annotation alone because CAZyme families can be broad and substrate specificity often requires experimental confirmation. Growth on defined substrates, substrate disappearance, acidification, metabolite profiling, and comparison with pathway-negative strains can establish whether the genome-predicted capacity is functional [48,112,113].

## Comparative and Population-Scale Probiogenomics

Comparative probiogenomics prevents single-genome storytelling. A single genome can identify candidate traits, but it cannot show whether those traits are common, rare, conserved, lineage-associated, mobile, or unusually combined. Pangenomes, core-gene phylogenomics, ANI, Mash distances, and trait overlays shift the question from what a strain encodes to how it differs from relevant relatives [45–48]. This context is critical because bacteriocin, EPS, AMR, carbohydrate-utilization, or stress-response loci may imply different priorities depending on whether they are conserved, accessory, niche-associated, or linked to mobile regions [38,45]. Comparative probiogenomics should also be interpreted through an evolutionary systems perspective. Probiotic-associated traits do not arise as isolated features; they are shaped by adaptive evolution, gene gain and loss, horizontal gene transfer, ecological niche adaptation, host-associated selection, microbial co-evolution with hosts, and fermentation-associated domestication. For example, carbohydrate-utilization pathways, stress-response systems, adhesion-related loci, bacteriocin clusters, EPS regions, and metabolite-production pathways may be conserved in some lineages but gained, lost, reorganized, or silenced in others. This evolutionary context helps determine whether a trait is stable and lineage-associated, recently acquired, mobile, niche-specific, or potentially costly to maintain. In addition, horizontal gene transfer networks and accessory-genome dynamics are particularly important because safety- and function-related traits may be carried by plasmids, prophages, integrative elements, or other MGEs. A bacteriocin cluster, AMR determinant, carbohydrate-utilization island, or stress-adaptation locus may therefore reflect both functional potential and evolutionary mobility. Similarly, host-associated evolution may shape traits related to mucosal interaction, bile adaptation, immune interaction, persistence, and substrate use, whereas fermentation-associated domestication may favor acid tolerance, carbohydrate metabolism, flavor-related pathways, phage resistance, or product-matrix adaptation. Evolutionary constraints should also be considered because not all predicted traits are beneficial in every context; some may impose metabolic costs, depend on specific substrates, or be expressed only under particular ecological conditions.

**Fig. 5.**
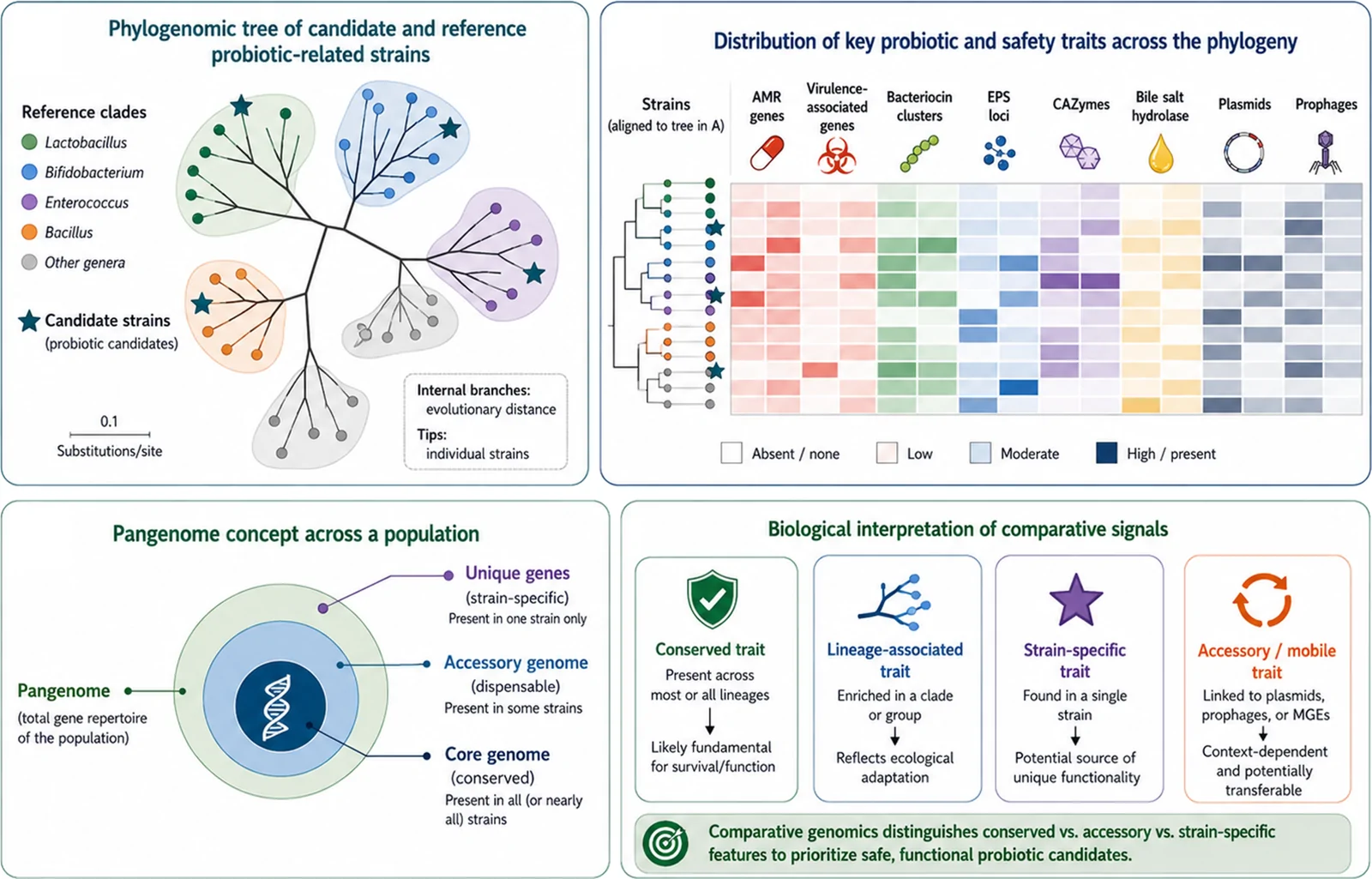
Comparative and population-scale probiogenomics. Phylogenomics, trait heatmaps, pangenome analysis, and biological interpretation of conserved, accessory, lineage-associated, and strain-specific signals provide context beyond single-genome annotation. This comparative layer helps distinguish stable candidate features from accessory or potentially mobile traits.

Recent genus-scale work on *Pediococcus* illustrates the value of this approach by integrating ANI, phylogenomics, pangenomes, AMR and virulence screening, BGCs, CAZymes, and probiotic-associated gene mining to show lineage-level functional diversity relevant to fermentation and probiotic applications [37]. Such work demonstrates how comparative genomics can move probiogenomic interpretation beyond isolated gene lists toward lineage-aware and evolution-aware strain prioritization. Trait-overlaid phylogenies and presence or absence heatmaps discourage species-level overgeneralization from a single *Lactiplantibacillus*, *Limosilactobacillus*, *Bifidobacterium*, *Enterococcus*, *Weissella*, or *Pediococcus* strain [114–116]. Thus, comparative genomics should be used to infer not only which traits are present but also how they evolved, how stable they are, whether they are mobile or niche-associated, and whether they are likely to function in the intended host, matrix, or application setting. Figure 5 summarizes how pangenome analysis, phylogenomic placement, and trait-overlay visualization provide the context needed to interpret safety- and function-related features beyond a single genome.

## Systems Biology, Multi-Omics, Artificial Intelligence, and Evidence Scoring

### Systems biology and metabolic modeling

Systems biology extends probiogenomics beyond genome annotation by asking how predicted genes, pathways, and strain traits operate within biological networks. Rather than interpreting candidate strains only through individual gene presence, systems-level probiogenomics considers pathway completeness, metabolic flux, regulatory control, microbial interactions, host context, and environmental conditions. This perspective is important because probiotic-associated functions including carbohydrate utilization, bile adaptation, antimicrobial activity, metabolite production, and host interaction are usually polygenic, condition dependent, and influenced by the surrounding microbial community. However, systems-level inference should be treated as a decision-support layer for hypothesis refinement and validation planning, not as direct evidence of probiotic function.

Genome-scale metabolic models, constraint-based reconstruction, and flux balance analysis can translate genome annotation into strain-level predictions of growth requirements, substrate utilization, metabolic bottlenecks, cross-feeding potential, and metabolite production. For candidate evaluation, these models can help prioritize substrates, prebiotics, product matrices, or fermentation conditions for testing. Their reliability depends on annotation quality, reaction curation, biomass assumptions, medium composition, uptake and secretion constraints, gap-filling choices, and available experimental data. Flux balance analysis usually predicts feasible steady-state behavior rather than actual growth dynamics, enzyme activity, metabolite accumulation, or host-context performance. Therefore, predicted fluxes or cross-feeding interactions should be validated using growth assays, substrate-consumption measurements, targeted metabolomics, isotope tracing where appropriate, or matched fermentation and host-associated models.

Network-based approaches can improve interpretation in community and host-associated contexts by predicting competition, cooperation, cross-feeding, niche overlap, and ecological compatibility between a candidate strain and resident microbiota. Nevertheless, co-occurrence, predicted complementarity, or modeled interaction does not establish persistence, colonization, direct interaction, or host benefit. Community-scale models require reliable strain-resolved genomes, abundance estimates, diet or medium composition, exchange-reaction assumptions, and validation under relevant ecological conditions. Similarly, regulatory-network inference generally requires transcriptomic data, perturbation experiments, promoter or motif evidence, transcription-factor annotation, time-series data, or other constraints; it cannot be inferred confidently from genome sequence alone. Host–microbe interaction models may connect microbial traits to epithelial interaction, immune signaling, barrier function, bile acid metabolism, or metabolite-mediated responses, but they require host-relevant systems and cannot infer clinical benefit from microbial genome content alone. Digital twin concepts remain prospective for most probiogenomic applications and should be framed as tools for scenario testing, study design, and personalized hypothesis generation rather than validated predictors of probiotic efficacy.

### Multi-omics integration

Multi-omics tests whether genome-predicted pathways are active under relevant conditions. Genomics identifies genes, pathways, loci, safety-relevant features, and comparative context; transcriptomics tests whether predicted genes are expressed under relevant stress, matrix, host-associated, or fermentation conditions; proteomics evaluates whether encoded proteins are produced; and metabolomics determines whether predicted pathways result in measurable products. Metagenomics or strain-resolved microbiome analysis can evaluate persistence, ecological effects, and community interactions, while pangenomics distinguishes conserved, accessory, and lineage-specific traits. Single-cell omics may add another layer by revealing strain- or cell-level heterogeneity that is masked in bulk analyses. When these layers are connected, probiogenomics moves from static genome annotation toward experimentally supported interpretation of pathway activity, strain behavior, phenotype, and host or application outcome [117,118].

The best use of multi-omics is hypothesis driven. A transcriptome designed around a genome-derived prediction can directly strengthen or weaken a mechanism, whereas broad descriptive profiling may be less informative [119]. For example, expression of a predicted bile-response module under bile stress is more informative than pathway presence alone, and detection of a predicted metabolite under the intended fermentation condition is more relevant than annotation of a biosynthetic gene in isolation [120]. Proteomic evidence can support whether adhesion proteins, stress proteins, transporters, or bacteriocin-associated proteins are produced, while metabolomic evidence can confirm whether predicted pathways result in biologically meaningful product accumulation. Recent clinical metabolomics work illustrates this point. In a secondary analysis of a randomized controlled trial, probiotic supplementation with *Lactobacillus helveticus* Rosell-52 and *Bifidobacterium longum* Rosell-175 was evaluated in relation to fecal short-chain fatty acids (SCFAs) and antidepressant treatment context. The probiotic effect on isovaleric acid differed according to antidepressant class, suggesting that metabolite-level outcomes may depend on host condition, co-medication, and intervention context [39]. This example shows why metabolomics is valuable for testing probiotic mechanisms, but also why metabolite changes should be interpreted as context-bound evidence rather than general proof of probiotic efficacy.

Moreover, multi-omics interpretation also depends on experimental design. Studies should report biological and technical replication, matched culture or host-associated conditions, sampling time, growth phase, stress exposure, product matrix, batch structure, normalization, statistical models, and cross-omics integration criteria. Temporal alignment is important because transcripts, proteins, metabolites, and phenotypes may change at different times. Batch effects or unmatched conditions can create misleading associations. Therefore, multi-omics integration should not be considered evidence of causality unless supported by appropriate controls, perturbation experiments, targeted validation, genetic confirmation, or matched phenotypic evidence.

### Artificial intelligence, machine learning, and explainability

Machine learning (ML) and artificial intelligence (AI) can support probiogenomics by ranking large genome collections, detecting latent genomic patterns, predicting functional potential, and integrating heterogeneous evidence. However, AI and ML applications in probiogenomics should be separated into established, emerging, and prospective uses because their evidence bases are not equivalent. More established applications include genome-based classification, AMR prediction, comparative trait prediction, genotype–phenotype association, and candidate ranking when curated labels and independent validation data are available. Supervised learning can support functional prediction or candidate prioritization when experimentally validated labels are available, whereas unsupervised learning can identify genome clusters, outliers, or candidate groups without predefined probiotic labels. Emerging approaches include genome embeddings, protein language models, graph neural networks, and other representation-learning methods that may capture sequence, protein-family, pathway, mobile-element, strain, microbiome, or host-associated patterns beyond simple gene presence or absence. Deep-learning models may also support functional annotation, antimicrobial peptide discovery, metabolic inference, genotype–phenotype association, and candidate ranking. Digital-twin approaches remain prospective and should be framed as tools for scenario testing, personalized hypothesis generation, and study design rather than validated predictors of probiotic efficacy. However, these approaches require careful validation. Current limitations include small training datasets, inconsistent probiotic definitions, publication bias, annotation bias, weak or insufficient labels, class imbalance, taxonomic imbalance, data leakage, overfitting, limited external validation, and restricted biological interpretability. Phylogenetic leakage is especially important because closely related strains in both training and test sets can inflate performance by allowing models to learn lineage structure rather than generalizable probiotic-associated biology. Random splits provide weak internal evidence; lineage-aware or cluster-aware splits better test generalization across related strains; species-level holdout validation evaluates transferability beyond the training taxa; and external validation using independently generated genomes and phenotypes provides the strongest evidence for candidate-prioritization models.

Explainable AI should be prioritized so that model outputs identify which genomic, functional, safety, or systems-level features drive a recommendation. Transparent prioritization should keep identity, genome quality, safety screening, functional potential, comparative context, and phenotype validation as separate evidence classes; a candidate with unresolved transferable AMR, toxin, or virulence signals should not rank highly because of attractive predicted functions alone. Thus, AI or ML should support specific decision points, such as comparator selection, trait prediction, uncertainty flagging, or validation prioritization, rather than replace safety-gated interpretation or operate as black-box ranking tools.

### Transparent scoring and evidence integration

**Fig. 6.**
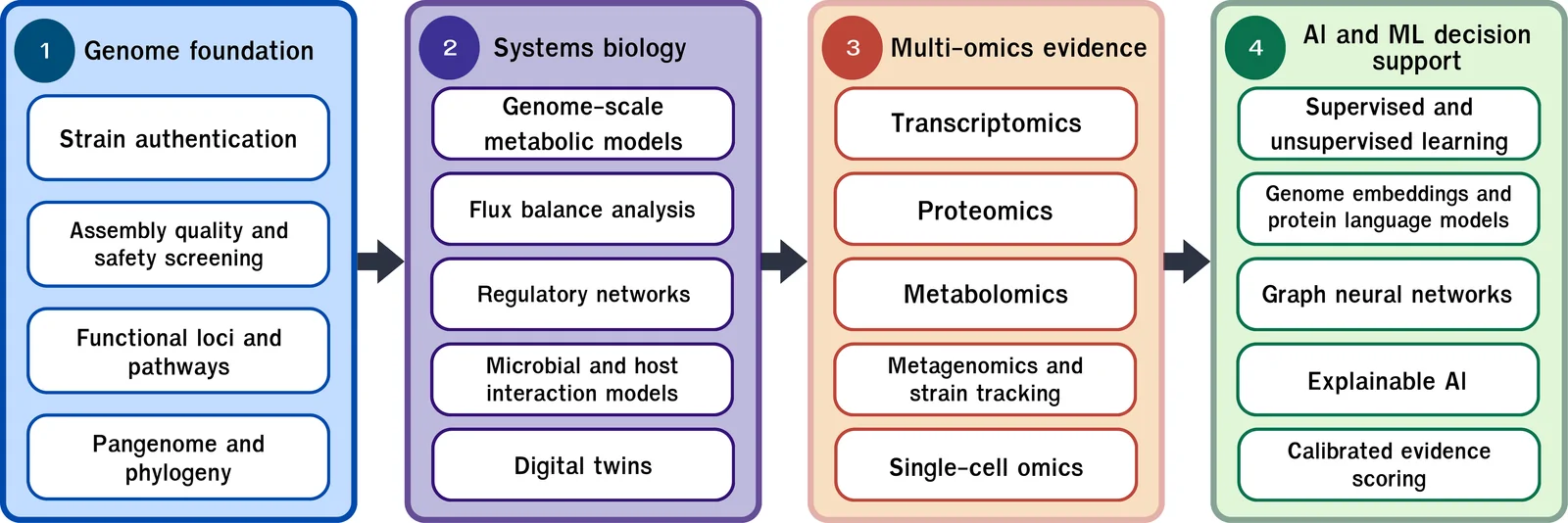
Systems biology, multi-omics, and AI-assisted probiogenomics. Genome-derived evidence is interpreted through metabolic, regulatory, microbial-interaction, and host-interaction models; tested across transcriptomic, proteomic, metabolomic, metagenomic, and single-cell layers; and integrated through transparent AI or ML decision support. High-confidence safety risks remain noncompensatory, whereas the resulting outputs guide candidate prioritization, matched validation, and intended-use interpretation.

Scoring systems can structure candidate prioritization, but they should not collapse qualitatively different evidence into a single unqualified score. A defensible scheme should first apply noncompensatory safety gates for high-confidence acquired AMR, toxin, virulence, or unresolved mobile-risk signals and then rank candidates that pass using separate domains for identity, genome quality, functional potential, comparative context, systems-level evidence, and validation status. Weights and thresholds should be predefined, sensitivity-tested, and externally validated where possible, with uncertainty flags and feature-level explanations. Missing data should not be treated as favorable evidence, and attractive functional predictions should not compensate for unresolved safety concerns. This explainable design can support reproducible advance, pause, exclude, or restrict-intended-use decisions while avoiding false precision [88,121–123]. Figure 6 summarizes how systems biology, multi-omics, and AI or ML contribute to the safety-gated decision framework.

## Phenotypic Validation and the Application-Dependent Evidence Continuum

**Fig. 7.**
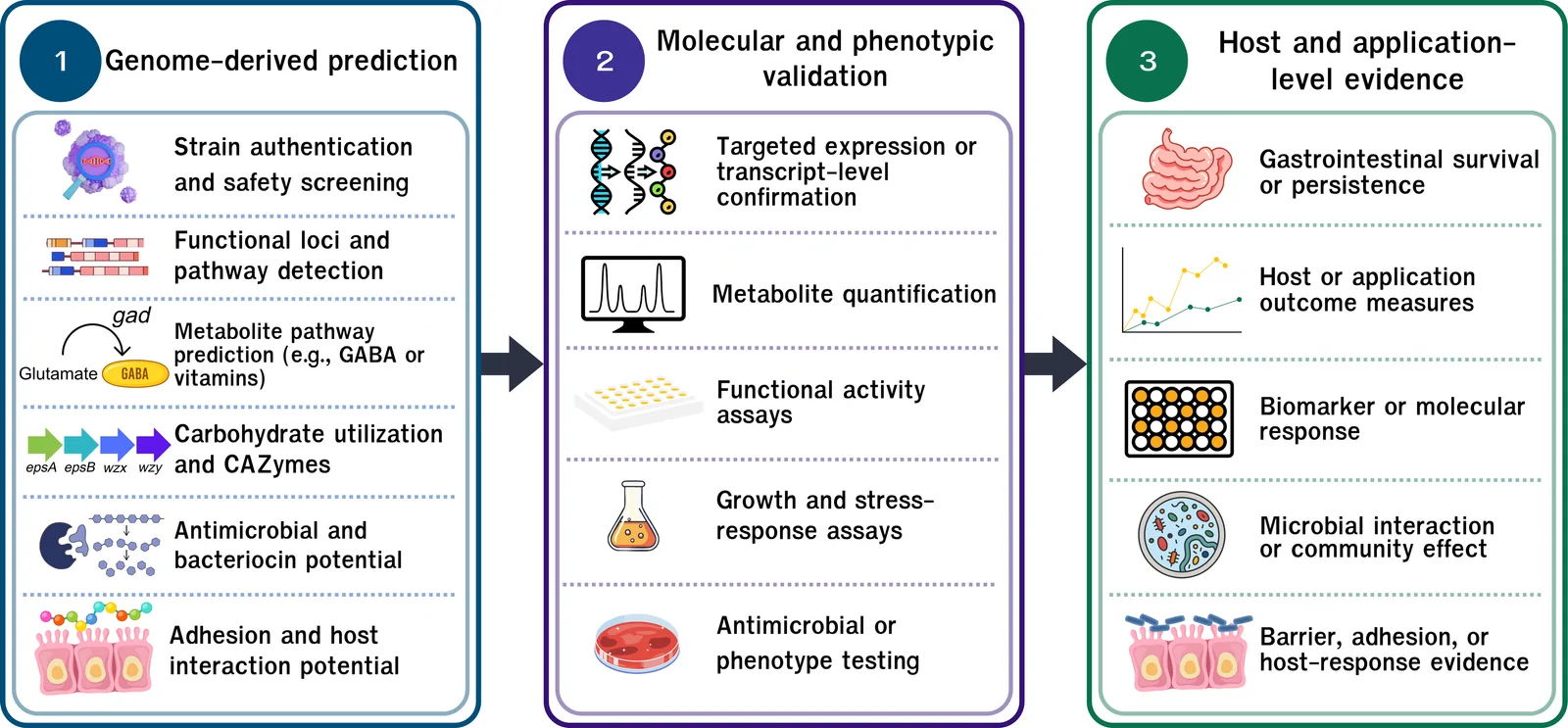
Application-dependent validation framework for probiogenomic predictions. Genome-derived predictions should be interpreted as a flexible evidence continuum, from predicted potential to molecular, phenotypic, host-, application-, or product-level support. Evidence requirements differ across food, feed, aquaculture, plant-associated, postbiotic, live-biotherapeutic, and clinical applications; therefore, claims should match the highest evidence level available.

Phenotypic validation converts genome-derived predictions into measured evidence and should be interpreted as an application-dependent evidence continuum rather than a mandatory linear sequence. Different applications may require different combinations of evidence levels depending on intended use, biological claim, and regulatory context. This continuum may include genomic prediction, transcriptomic confirmation, proteomic support, metabolite quantification, phenotypic characterization, community or host-model evidence, clinical or application-specific outcomes, and regulatory or product-level assessment. However, not every study requires all levels, and evidence may be generated in parallel, prioritized according to intended use, or omitted when irrelevant to the claim. A useful validation plan links each prediction to a specific assay and evidence level, such as BSH genes to bile-response or BSH activity assays, bacteriocin clusters to antimicrobial testing with appropriate controls, EPS loci to polymer characterization, GABA or vitamin pathways to targeted metabolite measurement, and carbohydrate-utilization modules to growth or metabolite assays on defined substrates [33]. Assay design should reflect the intended host, product matrix, dose range, exposure route, and application setting because laboratory phenotypes may not translate directly to food, feed, aquaculture, plant-associated, or clinical use. Thus, the appropriate evidence package depends on intended use, biological claim, regulatory jurisdiction, target host or population, product matrix, and claimed benefit. This evidence continuum is summarized in Table 6 and illustrated in Fig. 7.

**Table 6.** Application-dependent evidence continuum for probiogenomic validation and claim language. Evidence levels are not a mandatory linear sequence. The appropriate evidence package and claim language depend on intended use, biological claim, regulatory context, target host or population, product matrix, and strength of evidence.

Evidence level	Example evidence	Claim supported
Level 1. Genomic prediction	Genes, pathways, loci, AMR, virulence and toxin screening, CAZymes, BGCs, EPS loci, metabolite pathways, mobile-element context	Genome-predicted or putative potential
Level 2. Transcriptomic confirmation	RNA-seq or targeted expression analysis under acid, bile, stress, fermentation, matrix, host-associated, or substrate-specific conditions	Condition-dependent expression of predicted systems
Level 3. Proteomic support	Detection of stress proteins, adhesion proteins, transporters, enzymes, bacteriocin-associated proteins, or metabolic proteins	Protein-level support for predicted function
Level 4. Metabolite quantification	HPLC, LC-MS, GC-MS, targeted metabolite assays, SCFA profiling, GABA, vitamin or organic acid quantification	Biochemical activity or product accumulation under defined conditions
Level 5. Phenotypic characterization	Acid and bile survival, BSH activity, adhesion assays, antimicrobial assays with controls, EPS quantification, substrate-utilization assays, MIC testing, hemolysis, and biogenic amine assays	Measured phenotype under defined experimental conditions
Level 6. Community or host-model evidence	Strain persistence, microbiome shift, colonization model, epithelial or barrier assays, immune-response assays, animal models, and plant or aquaculture challenge models	Host-, matrix-, or ecosystem-relevant functional evidence
Level 7. Clinical, field, or application evidence	Human clinical trials, animal performance studies, aquaculture or plant field trials, food-fermentation performance, and product-stability studies	Application-specific benefit or performance evidence
Level 8. Regulatory and product-level evidence	Manufacturing control, dose justification, product consistency, safety package, target-population assessment, and regulatory submission or approval	Regulatory or claim-level support within a defined intended use

Validation priorities should also be application-specific. Human probiotic candidates may require host-interaction, persistence, safety, and clinical endpoint evidence; animal, aquaculture, and plant-associated candidates may require performance, pathogen-challenge, colonization, environmental-release, or ecological-impact assays; fermentation strains may require matrix-specific growth, metabolite safety, product consistency, and manufacturing-stability testing; postbiotic source strains may require product-composition and manufacturing-consistency evidence; and live biotherapeutic products may require defined strain identity, dose, stability, purity, potency, target-population safety, clinical evidence, and regulatory review. These application-specific evidence requirements are summarized in Table 4. When safety interpretation depends on uncertain genomic findings, targeted phenotypes such as MIC testing, hemolysis assays, biogenic amine measurement, toxin-relevant tests, or matrix-aware metabolite assays should be included [124]. Negative or discordant validation results are informative. A gene may be present but silent, a pathway incomplete, a bacteriocin cluster unexpressed, or a CAZyme profile insufficient to support growth on the proposed substrate [125,126]. Multi-omics can help resolve these discrepancies by testing whether predicted pathways are expressed, proteins are produced, and metabolites accumulate under relevant conditions. When genomic and phenotypic evidence conflict, interpretation should be re-evaluated in relation to genome quality, annotation confidence, pathway completeness, regulatory mechanisms, culture conditions, and assay design. Evidence language should distinguish prediction, association, measured activity, causation, and demonstrated benefit. Genomic or AI predictions indicate potential; statistical associations or omics correlations support hypotheses; measured phenotypes demonstrate activity under defined conditions; and causal or probiotic-benefit claims require mechanistic, host, application, or clinical evidence. A genome-supported and assay-confirmed trait can be described as a measured phenotype under defined conditions, but host-benefit claims require host-response, animal, clinical, or application-relevant evidence. For clinical translation or live biotherapeutic development, additional application-specific evidence and regulatory review are required, as summarized in Table 4 [9]. Therefore, probiogenomic conclusions should be written at the highest evidence level actually supported by the data, without treating prediction, association, correlation, phenotype, causation, and demonstrated benefit as equivalent.

## Proposed Workflow for Constructing Probiogenomic Research

A probiogenomic study can be organized as a sequence of auditable decision points. First, strain provenance should document the source, isolation method, culture history, storage, and stable identifier because traceability affects reproducibility and regulatory interpretation. Second, genome generation and quality control should report the sequencing platform, coverage, assembly method, genome size, contig number, N50, completeness, contamination, annotation pipeline, and data deposition before biological claims are made [127,128]. Third, strain authentication using ANI, standardized genome taxonomy, and phylogenomic placement should confirm the candidate’s identity and define appropriate comparator genomes. Fourth, the safety gate should evaluate AMR determinants, virulence-associated genes, toxins, plasmids, prophages, insertion sequences, MGEs, biogenic amine pathways, and other undesirable traits before functional promotion [14,129,130].

Only candidates that pass or resolve the safety gate should proceed to functional prioritization. At this stage, predicted traits should match the intended use and be linked to predefined validation assays. Comparative genomics can determine whether the candidate is distinctive or representative, while final claims should be labeled according to evidence level: taxonomic, genome-predicted, phenotypically validated, host-associated, or efficacy-supported. The workflow can support both prospective strain discovery and retrospective evaluation of published candidates. Although small studies may not require multi-omics or ML, they should still include identity confirmation, genome-quality assessment, safety screening, and at least one validation assay matched to a central prediction [49,131]. Table 7 summarizes the minimum reporting information needed to make probiogenomic studies traceable, reproducible, safety-gated, and explicitly linked to validation evidence.

**Table 7.** Minimum reporting checklist for probiogenomic studies. A candidate strain should not be promoted to a probiotic claim on the basis of genome data alone. Identity, safety, function, and benefit operate at different evidence levels and should be discussed as such [1,2,6].

Reporting domain	Minimum information to report	Why it matters
Strain provenance	Source, isolation context, host or matrix, isolation method, culture history, storage condition, stable strain ID, and culture-collection or product documentation where available.	Enables traceability, biological interpretation, comparator selection, and reproducibility.
Intended use and application context	Proposed use, target host or population, product type, exposure route, dose range if known, product matrix, environmental-release context, and regulatory pathway.	Safety and validation requirements differ for human, animal, aquaculture, plant-associated, fermentation, dietary supplement, and live biotherapeutic applications.
Sequencing and assembly	Sequencing platform, library type, read type, read depth or coverage, assembler, assembly mode, polishing strategy, circularization status, plasmid-resolution approach, genome size, GC content, contig number, N50, completeness, contamination, and accession numbers.	Determines whether safety and functional traits can be interpreted only as gene presence or absence or in genomic context.
Genome quality	Completeness, contamination, number of contigs, N50, genome size, GC content, ambiguous bases if reported, evidence of possible mixed culture, and quality-assessment tool and version.	Defines whether the assembly is reliable enough for taxonomy, AMR screening, MGE interpretation, and functional prediction.
Taxonomic authentication	ANI values, GTDB, GTDB-Tk or equivalent classification, type-strain comparison, closest reference genomes, phylogenomic placement, database version, nomenclature used, species-boundary criteria, and unresolved taxonomy if present.	Prevents misclassification and clarifies safety expectations, comparator selection, and species- or strain-level claims.
Annotation workflow	General annotation tool, specialized annotation tools, software versions, database releases, parameters, gene-calling approach, and manual-curation steps.	Allows reanalysis and reduces overinterpretation of automatic gene labels.
Safety screening	AMR genes, resistance-associated mutations, virulence-associated genes, toxin genes, plasmids, prophages, insertion sequences, other MGEs, biogenic amine pathways, undesirable metabolic traits, detection thresholds, and safety-call criteria.	Ensures safety screening precedes functional promotion.
AMR interpretation	Database or tool used, identity and coverage thresholds, gene completeness, intrinsic versus acquired interpretation, chromosomal versus mobile context, neighboring genes, and MIC testing if relevant.	Distinguishes intrinsic, acquired, mobile, ambiguous, and phenotype-supported resistance.
Mobile-element context	Plasmid replicons, prophage regions, insertion sequences, transposons, integrative elements, contig context, circularization evidence, and whether key genes are linked to mobile regions.	Indicates transferability, stability, and uncertainty of safety- or function-related traits.
Functional trait mining	Specific predicted traits, gene and pathway names, pathway completeness, gene neighborhood, relevant databases, comparative context, and matched assay.	Converts gene lists into testable hypotheses rather than unsupported probiotic claims.
Comparative context	Comparator-genome selection, inclusion or exclusion criteria, pangenome method, ANI or Mash distance method, phylogenomic method, trait matrix, and lineage or niche interpretation.	Clarifies whether traits are conserved, accessory, lineage-specific, mobile, niche-associated, or strain-specific.
Phenotypic validation	Validation assays performed, assay conditions, controls, endpoints, biological replicates, statistical approach if applicable, and whether assays directly test genome-derived predictions.	Converts genome-predicted potential into measured evidence under defined conditions.
Evidence-level and claim language	Explicit distinction among genomic prediction, statistical association, omics correlation, measured phenotype, causal evidence, host- or application-level evidence, and demonstrated probiotic benefit.	Prevents overinterpretation and keeps claims aligned with the actual strength of evidence.
Decision logic	Criteria for advance, pause, exclude, or restrict intended use; uncertainty flags; safety-gate outcome; functional-prioritization rationale; and validation-priority ranking.	Makes safety-gated prioritization auditable.
Data and code availability	Genome accession numbers, raw-read accessions if available, supplementary evidence tables, tool versions, databases, scripts, workflow files, containers or environments, and repository links.	Supports reproducibility, benchmarking, and future reanalysis.
Negative and ambiguous findings	Negative database screens, absent pathways, incomplete loci, negative or inconclusive assays, ambiguous hits, unresolved taxonomy, unresolved mobile context, and reasons for pause, exclusion, or restriction.	Reduces publication bias and defines the limits of safety and functional interpretation.

### Illustrative application scenarios

The scenarios below illustrate how the safety-gated workflow can be adapted to taxon and intended use. A Lactobacillaceae or Bifidobacterium candidate with resolved taxonomy, acceptable genome quality, no high-confidence acquired AMR gene, no concerning toxin or virulence locus, and no unresolved mobile safety signal may advance to matched validation of predicted traits such as BSH activity, EPS production, GABA synthesis, bacteriocin potential, or carbohydrate utilization [31,132]. By contrast, an *Enterococcus*-like candidate with attractive functional potential but a complete acquired AMR gene, cytolysin-like locus, high-confidence virulence region, or plasmid-associated safety concern should pause, be restricted, or be excluded depending on mobility, phenotype, intended use, and regulatory relevance. A spore-forming or food-associated candidate may be promising for fermentation or delivery but may still require additional toxin, sporulation, persistence, environmental-release, and matrix-specific metabolite assessment [133]. These scenarios emphasize that the framework is not a pass or fail checklist; the same predicted trait may support advancement in one context, require additional testing in another, or become irrelevant when a higher-priority safety concern remains unresolved.

### Worked examples of safety-gated decision-making

The following hypothetical examples show how the safety-gated probiogenomic framework converts genome-mining results into auditable decisions rather than simple lists of favorable and unfavorable genes.

Example 1: Functional potential with unresolved safety evidence. A candidate strain contains predicted bile-response genes, EPS-associated genes, CAZymes, and a bacteriocin-like cluster. A conventional genome-mining workflow might prioritize the strain based on these favorable features. However, the same genome also contains an ambiguous virulence-associated hit and an AMR determinant with unresolved plasmid context. Under the safety-gated framework, the strain would be classified as “pause” rather than “advance” until the findings are resolved through manual curation, improved assembly, taxon-aware comparison, or targeted phenotypic and mobility testing. The safety concern does not establish hazard or transferability, but its uncertainty prevents functional predictions from overriding unresolved safety evidence.

Example 2: Predicted metabolic pathway with progressive validation. A strain contains a genome-predicted glutamate decarboxylase pathway and is therefore considered a potential GABA producer. Genome evidence alone supports only a functional hypothesis. Transcriptomic evidence, targeted GABA quantification by HPLC or LC-MS, and matrix-specific testing can progressively strengthen the claim from predicted potential to demonstrated activity under defined conditions. However, a broader probiotic-benefit claim would require appropriate host, animal, clinical, or application-specific evidence.

These examples show that the framework changes the decision process rather than simply reorganizing genome-mining outputs: Unresolved safety evidence can trigger a pause despite favorable functional features, whereas functional predictions require progressively matched evidence before stronger claims are made.

## Computational Reproducibility, Benchmarking, and Scalable Implementation

Probiogenomics is most useful when it produces reproducible decision outputs rather than narrative claims that a strain contains beneficial genes and lacks obvious hazards. A useful output should be a traceable analytical object that includes genome inputs, accession identifiers, software versions, database releases and accession dates where applicable, thresholds, intermediate files, manual-curation notes, decision rules, and validation recommendations. At minimum, authors should provide a strain-level metadata table, genome-quality table, safety-call matrix, functional-trait matrix, comparator-genome list, and decision log explaining why each strain was advanced, paused, excluded, or selected for validation [134]. A reproducible workflow should begin with stable identifiers and machine-readable metadata, including isolate name, source, sequencing and assembly accessions, taxonomic assignment, genome-quality statistics, intended use, and analysis date [135]. Benchmarking remains difficult because candidate-probiotic studies often use different AMR databases, virulence databases, assembly methods, annotation pipelines, and functional-mining tools [136,137]. Therefore, benchmark-ready workflows should record not only final calls but also identity and coverage thresholds, gene completeness, contig length, neighboring genes, predicted mobility, manual-curation status, negative findings, and uncertainty flags. Workflow managers and containerized environments, including Nextflow, Snakemake, Docker, Singularity and Apptainer, and Conda, can improve reproducibility by preserving software dependencies, execution order, parameter settings, and intermediate outputs [135,138]. Scalable implementation is increasingly important as public genome collections grow. Pangenome analysis, Mash-based screening, ANI clustering, trait matrices, and safety dashboards can convert large genome sets into ranked candidate lists. However, ranking systems should remain interpretable: Safety-relevant findings should be exclusionary or heavily weighted, whereas functional predictions should increase priority only when pathway completeness, comparative context, and validation feasibility are coherent. Machine-learning models trained on weak probiotic labels may learn taxonomic popularity, publication bias, or commercial history rather than causal probiotic traits [122]. A computationally responsible model should therefore keep safety features, functional predictions, systems-level evidence, experimental validation, and intended use as separate evidence classes [88].

The long-term goal is a community-level probiogenomic knowledge graph or evidence matrix linking genomes, metadata, safety calls, predicted traits, validation assays, negative findings, and application contexts. Such resources would make probiogenomics more comparable across laboratories and more useful for automated candidate discovery, regulatory review, and computational biotechnology applications. Table 8 provides a workflow-output checklist covering strain metadata, assembly quality, taxonomy, annotation, safety screening, functional mining, comparative context, prioritization logic, validation planning, and data or code availability.

**Table 8.** Computational reproducibility and workflow-output checklist for probiogenomic studies

Workflow component	Minimum computational record	Why it matters	Recommended output
Strain and metadata	Stable strain ID, source, accession, analysis date, intended use	Enables traceability and re-analysis	Machine-readable metadata table
Genome assembly	Sequencing platform, assembler, coverage, contig count, N50, genome size, circularization status where relevant	Defines reliability of AMR, plasmid, prophage, and cluster calls	Assembly-quality report
Genome quality	Completeness, contamination, ambiguous bases, possible mixed culture	Prevents false safety and function inference	Quality-control summary
Taxonomy	ANI, GTDB-Tk result, comparator genomes, thresholds	Establishes correct biological frame	Taxonomic-authentication table
Annotation	Tool versions, databases, parameters	Annotation changes with tool or database updates	Versioned annotation files
Safety screening	AMR, virulence, toxin, plasmid, prophage, MGE, biogenic amine calls	Defines advance, pause or exclude decisions	Safety-gate matrix
Functional mining	Trait predictions, pathway completeness, gene context	Converts genes into hypotheses	Trait-prediction table
Comparative context	Comparator selection, pangenome method, phylogeny, trait matrix	Distinguishes conserved *vs*. strain-specific signals	Comparative-genomics dashboard
Transcriptomics	Growth condition, stress exposure, sampling time, replicates, normalization method, differential-expression criteria	Tests whether genome-predicted traits are expressed under relevant conditions	Expression matrix and differential-expression report
Proteomics	Culture condition, protein extraction method, database used, peptide or protein identification thresholds	Supports whether predicted proteins are produced under relevant conditions	Protein-detection and abundance table
Metabolomics	Matrix, substrate, growth condition, analytical platform, standards, normalization and quantification method	Confirms whether predicted metabolic pathways produce measurable compounds	Targeted or untargeted metabolite profile
Metagenomics or strain tracking	Sample type, sequencing method, strain-resolution approach, abundance threshold, persistence criteria	Evaluates strain persistence, community effects, and ecological behavior	Strain-tracking and community-analysis report
Machine-learning analysis	Feature definitions, training labels, class balance, feature selection, validation design, performance metrics, external validation where available	Prevents black-box ranking, label bias, overfitting, and poor biological interpretability	Model report with explainability outputs
Prioritization logic	Scoring rules, exclusion rules, uncertainty flags	Makes ranking auditable	Decision log
Validation planning	Prediction-to-assay mapping and evidence level	Links computation to experiments	Validation-priority table
Reproducibility	Code, software environment, containers, workflow manager if available	Enables independent re-analysis	GitHub, Zenodo or OSF archive

### False positives, false negatives, and uncertainty reporting

False-positive and false-negative results are unavoidable in probiogenomic analysis and should be reported as part of the evidence model. False positives may result from low-specificity annotations, short conserved domains, permissive identity or coverage thresholds, contamination, chimeric contigs, pseudogenes, fragmented genes, or database entries derived from distant taxa [66]. This is especially relevant for virulence and functional-trait databases, where surface, adhesion, stress-response, transporter, or iron-acquisition genes may be annotated as risk- or probiotic-associated despite context-dependent biological roles [78,81]. False negatives may occur when databases are incomplete, genes are divergent, assemblies fragment AMR, toxin, bacteriocin, EPS, or metabolic loci, or gene prediction fails because of sequencing errors, frameshifts, or pseudogenes [139,140]. Some phenotypes also depend on mutations, regulation, copy number, expression, growth conditions, substrate availability, host context, or product matrix rather than simple gene presence or absence. Therefore, absence of a database hit should be reported as no high-confidence hit detected under the specified tools, database versions, thresholds, and genome quality, rather than as proof of absence or safety. Ambiguous AMR, virulence, toxin, mobile-element, or functional-trait calls should remain visible in final decision outputs, with confidence information such as identity, coverage, gene completeness, contig context, taxonomic relevance, and manual or phenotypic validation status.

Uncertainty reporting should also extend to multi-omics and machine-learning analyses. For multi-omics studies, authors should report sample metadata, growth conditions, stress exposure, sampling time, batch structure, normalization methods, statistical models, and data-processing parameters [141]. For machine-learning analyses, authors should report feature definitions, training labels, class balance, feature-selection strategy, validation design, performance metrics, external validation where available, code availability, and interpretability outputs [121].

### Limitations of the safety-gated probiogenomic framework

Despite its potential, the safety-gated probiogenomic framework is a decision-support approach for candidate prioritization, not a validated universal safety model. Its conclusions remain conditional on genome quality, taxonomic resolution, database coverage, analytical thresholds, intended use, and available validation evidence. Incomplete or biased databases may generate false-positive or false-negative safety calls, while fragmented assemblies, contamination, mixed cultures, misassemblies, and unresolved plasmid or prophage structures can affect AMR, virulence, toxin, MGE, and functional-trait interpretation. Taxonomic bias is also important because safety and functional databases are unevenly represented across genera, species, and strain lineages.

A second limitation is genotype–phenotype discordance. Gene presence does not establish expression, biological activity, metabolite accumulation, transferability, or host benefit, while absence of a database hit does not prove absence of risk. Predicted mobile-element associations are also uncertain because plasmid-like contigs, prophage predictions, transposase proximity, or MGE-adjacent loci do not by themselves establish mobility or horizontal transfer. Similarly, AI- or ML-assisted prioritization remains limited by weak labels, small training datasets, class and taxonomic imbalance, phylogenetic leakage, publication bias, overfitting, and limited external validation.

Finally, regulatory heterogeneity limits generalization. Safety expectations, evidence requirements, claim language, and acceptable uncertainty differ across food, feed, dietary supplement, live biotherapeutic, aquaculture, plant-associated, fermentation, and postbiotic applications. Therefore, the framework should preserve uncertainty, identify risks early, guide validation, and align claims with evidence levels rather than replace phenotypic testing, causal experiments, application-specific assessment, or regulatory decision-making.

## Challenges and Future Perspectives

The main challenge in probiogenomics is not sequencing availability, but interpretation, evidence integration, and reproducibility. Fragmented assemblies, incomplete databases, taxonomic and annotation bias, inconsistent thresholds, changing taxonomy, sparse phenotype labels, and weak metadata can affect manual review, comparative analyses, and machine-learning models. Interpretation of plasmids, prophages, and other MGEs is further limited by assembly quality and prediction uncertainty, while genotype–phenotype discordance can complicate safety and functional conclusions. A reassuring genome screen is therefore conditional on genome quality, database version, analytical thresholds, and intended-use context. Another persistent challenge is the underreporting of negative or uncertain evidence, including strains excluded because of AMR, virulence-like hits, poor assembly quality, weak tolerance phenotypes, unstable mobile elements, or absent metabolite production. Future datasets should link high-quality genomes with assay protocols, measured phenotypes, host or matrix context, negative or inconclusive results, and reasons for exclusion, thereby making AI-assisted prioritization more transparent and less dependent on publication history or reputation-based labels [122,123].

Future probiogenomics should also move toward systems-level, evidence-linked, and application-aware implementation. Long-read and hybrid assemblies, curated strain databases, standardized safety panels, richer phenotype metadata, multi-omics, genome-scale metabolic models, microbial interaction networks, and interpretable AI or ML approaches can improve candidate prioritization. However, these approaches remain dependent on data quality, appropriate experimental validation, and context-specific interpretation. Terminology and claim language must remain precise. Candidate probiotics, starter cultures, live biotherapeutic candidates, postbiotic sources, commensal isolates, and beneficial microbes represent different evidence and application contexts. Regulatory requirements also vary across intended uses and jurisdictions, limiting the applicability of universal decision thresholds. Genome data should reduce uncertainty, expose risk earlier, guide validation, and support decision-making, but should not replace safety testing, manufacturing controls, host-response experiments, efficacy evidence, or regulatory assessment. Thus, safety-gated probiogenomics should be viewed as an evidence-integration framework rather than a validated universal model of safety or probiotic efficacy.

## Conclusion

Probiogenomics is best understood as a genome-resolved, decision-oriented framework for evaluating and prioritizing microbial candidates for defined applications rather than a genome-only route to probiotic claims. Its main value is not the introduction of entirely new bioinformatic modules, but the organization of established genome-based practices into a transparent and auditable decision system. In this safety-gated probiogenomic framework, strain provenance, genome quality, taxonomic authentication, and safety-relevant screening should precede functional promotion. AMR genes, virulence- and toxin-associated loci, plasmids, prophages, MGEs, undesirable metabolic pathways, and genotype–phenotype contradictions should be interpreted through evidence categories that consider detection, biological plausibility, mobility, phenotypic support, confidence, and intended-use relevance, distinguishing high-confidence risk signals from ambiguous or method-limited findings. Importantly, the absence of detected AMR, virulence, toxin, or mobile-element signals should be reported as no high-confidence risk signal detected under the specified tools, databases, thresholds, and genome quality, rather than as proof of complete safety.

The framework also emphasizes that safety and functional evaluation are context dependent and intended-use specific. Safety interpretation, functional relevance, validation endpoints, and acceptable claim language differ across human probiotics, animal feed probiotics, aquaculture probiotics, plant-associated beneficial microbes, fermentation or starter cultures, dietary supplements, and live biotherapeutic products. Therefore, candidate decisions such as advance, pause, exclude, or restrict intended use should consider the target host or population, dose, route of exposure, product matrix, manufacturing process, environmental-release potential, phenotypic safety evidence, and jurisdiction-specific regulatory pathway. This modular logic prevents attractive genome-predicted functions from overriding unresolved safety concerns. Functional trait mining should support pathway- and systems-level hypothesis generation after safety-gated interpretation, rather than being treated as direct evidence of probiotic benefit. Predicted traits such as acid and bile tolerance, adhesion, EPS production, bacteriocin synthesis, GABA or vitamin production, carbohydrate utilization, or stress response require matched validation before being described as phenotypes. Multi-omics can strengthen interpretation by testing whether predicted pathways are expressed, proteins are produced, or metabolites accumulate under relevant conditions, while ML and scoring systems can support prioritization when appropriately validated, interpretable, and subordinate to safety and application-specific evidence. These approaches should be used as decision-support tools, not as substitutes for phenotypic testing or host-benefit evidence.

For the field to mature, probiogenomic studies should move beyond gene lists toward reproducible and benchmark-ready workflows. Software versions, database releases and accession dates where applicable, thresholds, comparator selection, contig context, mobile-element localization, negative and uncertain findings, decision logic, and validation outcomes should be reported in machine-readable formats whenever possible. Workflow managers and containerized environments can further improve reproducibility by preserving software dependencies, execution order, parameters, and intermediate outputs. However, the safety-gated probiogenomic framework should be regarded as an evidence-integration and decision-support framework, not a validated universal model of safety or probiotic efficacy. By preserving uncertainty, exposing risk early, separating predicted genetic potential from validated evidence, and linking genome analytics to an application-dependent evidence continuum and intended-use-specific interpretation, the safety-gated probiogenomic framework can support more responsible, reproducible, and application-aware candidate microbial development.
